# Vpu Exploits the Cross-Talk between BST2 and the ILT7 Receptor to Suppress Anti-HIV-1 Responses by Plasmacytoid Dendritic Cells

**DOI:** 10.1371/journal.ppat.1005024

**Published:** 2015-07-14

**Authors:** Mariana G. Bego, Édouard Côté, Nick Aschman, Johanne Mercier, Winfried Weissenhorn, Éric A. Cohen

**Affiliations:** 1 Institut de Recherches Cliniques de Montréal (IRCM), Montreal, Quebec, Canada; 2 Université Grenoble Alpes, Unit of Virus Host Cell Interactions (UVHCI), CNRS, UVHCI, Grenoble, France; 3 Department of Microbiology, Infectiology and Immunology, Université de Montréal, Montreal, Quebec, Canada; University of Pennsylvania School of Medicine, UNITED STATES

## Abstract

Plasmacytoid dendritic cells (pDCs) constitute a major source of type-I interferon (IFN-I) production during acute HIV infection. Their activation results primarily from TLR7-mediated sensing of HIV-infected cells. However, the interactions between HIV-infected T cells and pDCs that modulate this sensing process remain poorly understood. BST2/Tetherin is a restriction factor that inhibits HIV release by cross-linking virions onto infected cell surface. BST2 was also shown to engage the ILT7 pDC-specific inhibitory receptor and repress TLR7/9-mediated IFN-I production by activated pDCs. Here, we show that Vpu, the HIV-1 antagonist of BST2, suppresses TLR7-mediated IFN-I production by pDC through a mechanism that relies on the interaction of BST2 on HIV-producing cells with ILT7. Even though Vpu downregulates surface BST2 as a mean to counteract the restriction on HIV-1 release, we also find that the viral protein re-locates remaining BST2 molecules outside viral assembly sites where they are free to bind and activate ILT7 upon cell-to-cell contact. This study shows that through a targeted regulation of surface BST2, Vpu promotes HIV-1 release and limits pDC antiviral responses upon sensing of infected cells. This mechanism of innate immune evasion is likely to be important for an efficient early viral dissemination during acute infection.

## Author Summary

Plasmacytoid dendritic cells (pDCs) produce large quantities of type I interferon (IFN-I) upon stimulation by many viruses, including HIV. Their activation is very effective following cell contacts with HIV-1-infected CD4+ T cells. We investigated whether HIV-1 could regulate the antiviral responses of pDCs triggered upon sensing of infected cells. We show that HIV-1 suppresses the levels of IFN-I produced by pDCs through a process that requires expression of the Vpu accessory protein in virus-producing cells. A well-described role of Vpu is to promote efficient HIV-1 production by counteracting BST2, a host factor that entraps nascent viral particle at the cell surface. Apart from its antiviral activity, BST2 was reported to inhibit IFN-I production by pDCs through binding and activation of the ILT7 pDC-specific inhibitory receptor. Our results reveal that through a highly sophisticated targeted regulation of BST2 levels at the surface of infected cells, Vpu promotes HIV-1 release and limits IFN-I production by pDCs via the negative signaling exerted by the BST2-ILT7 pair. Overall, this study sheds light on a novel Vpu-BST2 interaction that allows HIV-1 to escape pDC antiviral responses. This modulation of pDC antiviral response by HIV Vpu may facilitate the initial viral expansion during acute infection.

## Introduction

Plasmacytoid dendritic cells (pDCs) are a distinct subset of DCs that exhibit a unique ability to secrete high amounts of interferons and other cytokines in response to viruses. Even though they constitute less than 1% of the total cell content of peripheral blood in humans, they are considered a primary source of type-I IFN (IFN-I) for antiviral responses. Hence, pDCs represent the first line of defense against viral infections, and as such, serve as a vital link between innate and adaptive immunity. Detection of virus infection by pDCs is mediated through recognition of viral nucleic acids, including single-stranded RNA (ssRNA) and double-stranded DNA containing unmethylated CpG motifs by the Toll-like receptor 7 (TLR7) and 9 (TLR9) endosomal sensors. Activation of TLR7/9 induces signaling events that ultimately lead to the stimulation of IFN genes through IRF7 and pro-inflammatory cytokines genes via NF-κB [1].

The role of pDCs during HIV infection appears to be complex [2]. pDCs are activated in HIV and SIV infection and are rapidly depleted from blood, coinciding with their redistribution to lymph nodes and mucosal tissues [3] where they are largely responsible for the IFN-I being produced during acute infection [4]. In addition, pDCs may be chronically stimulated during HIV infection and a continuing source of IFN-I, a feature that seems central to the immune activation and the CD4+ T cell loss during pathogenic infection [2,5]. pDCs express the primary HIV receptor, CD4, as well as the main co-receptors, CXCR4 and CCR5, and as such support entry of X4 and R5 strains of HIV [6]. Upon sensing HIV-1, pDCs produce IFN-I and other cytokines, and undergo phenotypic activation [7–9]. Although high concentrations of purified HIV virions are capable of inducing IFN-I from pDCs, HIV-infected CD4+ T cells are much more effective at stimulating these IFN-producing cells (10-100-fold relative to cell-free virus) given their ability to establish cell contacts with them [6,8,10]. Thus, potent recognition of cell-associated HIV by pDCs may represent an important host strategy to overcome the poor detection of cell-free virions.

HIV-infected cells are sensed by pDCs through a process that involves endocytosis and fusion of virions that are transferred across cell contacts. Once fusion is completed, the ssRNA genome is believed to gain access to endosomes where recognition occurs in a large part through TLR7. This pathway of recognition of cell-associated HIV appears to differ from detection of cell-free HIV, in which there is a requirement for attachment and endocytosis but not for viral fusion [6,8,11]. Whether cell-free HIV-1 and cell-associated HIV-1 are recognized by separate pathways that converge on TLR7 remains unclear. Since efficient sensing involves cell contacts between pDCs and infected cells, it is conceivable that viral proteins and/or host factors expressed at the surface of HIV-producing CD4+ T cells might modulate pDC sensing or/and antiviral responses. Indeed, the HIV envelope glycoprotein gp120 was found to inhibit TLR9-mediated antiviral responses most likely by interacting with the pDC specific inhibitory receptor, BDCA-2 [12,13]. However, the interactions between HIV-infected T cells and pDCs that modulate TLR7-mediated pDC sensing and the resulting antiviral responses remain poorly understood.

BST2/Tetherin (also known as CD317) is a glycosylated type II integral membrane protein that is induced by IFN-I (for review, see [14]). The dimeric protein has a distinctive topology as it contains two membrane anchors. It consists of a short N-terminal cytosolic tail, a transmembrane (TM) domain, an elongated extracellular coiled-coil [15] and a glycophosphatidyl-inositol (GPI)-linked membrane anchor present at the C-terminus [16]. BST2 was found to strongly inhibit the release of HIV and other enveloped viral particles [17,18] and to impede cell-to-cell HIV transmission [19,20], although this notion has been challenged [21]. BST2 restricts HIV release through a direct “tethering” mechanism whereby the protein cross-links progeny virions to cell membranes by a process that requires both membrane anchors [22]. Interestingly, apart from its direct antiviral activity, BST2 has also been reported to act as the ligand of immunoglobulin-like transcript 7 (ILT7, also known as LILRA4 and CD85g), a receptor preferentially expressed at the surface of human pDCs [23]. ILT7 is an inhibitory receptor that controls inflammation by modulating the production of IFN-I and other pro-inflammatory cytokines by pDCs [24]. Indeed, binding of BST2 to ILT7 was found to negatively regulate activation of the two key pDC sensors, TLR7 and TLR9, hence controlling IFN-I secretion [25].

The prototypical HIV-1 molecular clone pNL4.3 and pandemic HIV-1 group M strains counteract the antiviral activity of BST2 via the viral protein U (Vpu) accessory protein. Vpu was found to enhance the release of progeny virions by downregulating BST2 from the cell surface of infected cells [17,18], although enhanced viral release in the absence of BST2 downregulation has been reported [26]. In the absence of Vpu, a large number of fully formed and mature HIV-1 particles are entrapped by BST2, thus forming large clusters of virions at the cell surface. These viral clusters could possibly occlude BST2 regulatory interactions as well as become potent targets for immune sensing by pDCs. We therefore hypothesized that BST2 antagonism by Vpu might modulate the ILT7 inhibitory signal that regulates TLR7/9-mediated production of IFN-I in activated pDC. In this study, we tested this hypothesis.

## Results

### Vpu suppresses the production of IFN-I during innate sensing of HIV-1 infected cells by PBMCs

**Fig 1:**
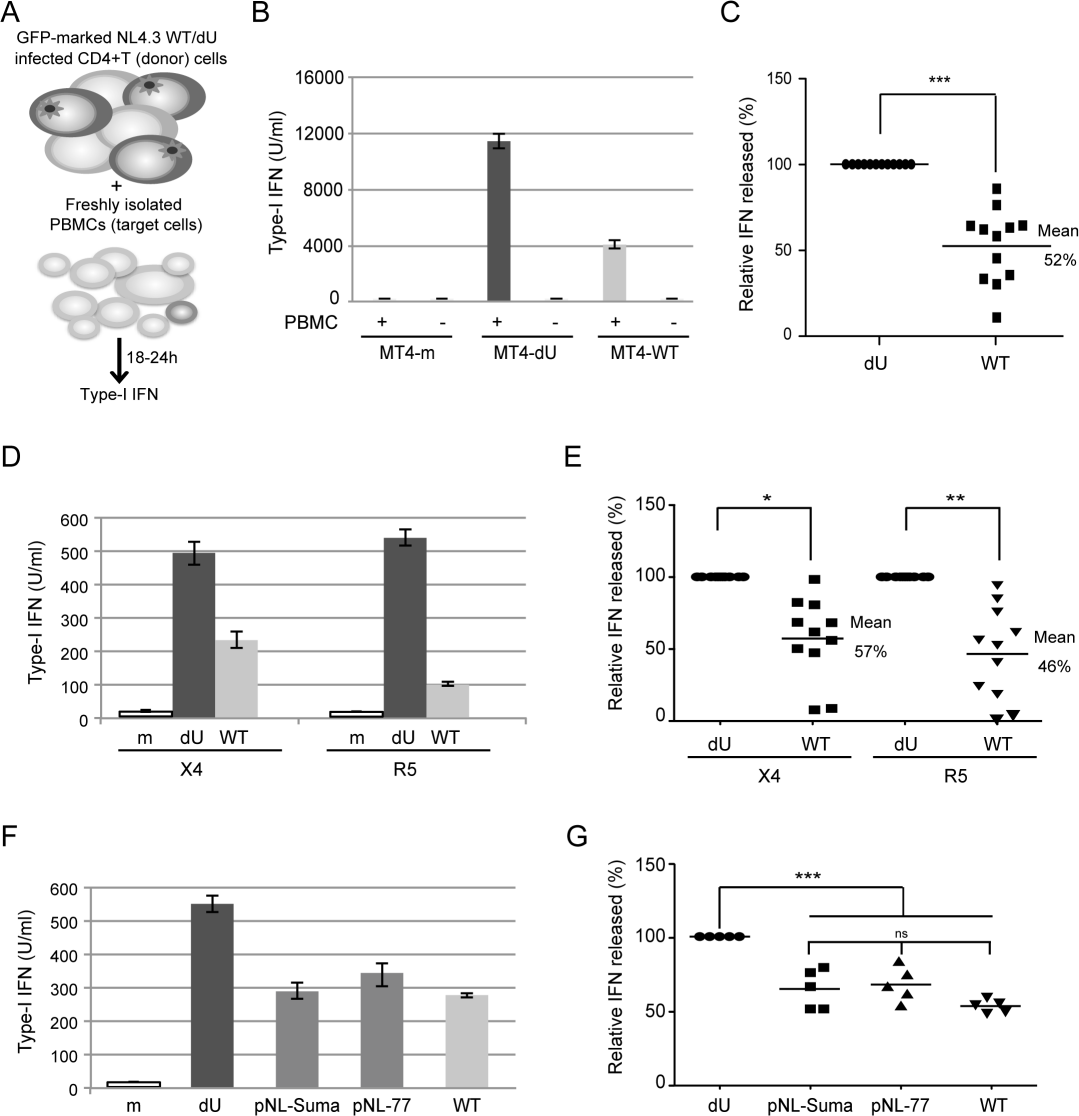
Vpu controls IFN-I production by PBMCs following contact with HIV-infected CD4+ T cells. **(A)** Schematic representation of the experimental system. WT or dU HIV-infected T cells are co-cultured with freshly isolated PBMCs and levels of bioactive IFN-I released in supernatants are measured 18–24h later. **(B-C)** MT4 cells were mock-infected, or infected with GFP-marked NL4.3 WT or dU viruses for 48 h, and co-cultured with PBMCs. After 24 h of co-culture, levels of IFN-I released in supernatants were measured. A representative example of absolute levels **(B)** or relative percentage **(C)** of IFN-I detected after co-culture of WT or dU HIV-1-infected MT4 donor cells with PBMCs (n = 12) are shown. **(D-E)** Primary CD4+ T cells were mock-infected, or infected with GFP-marked NL4.3 (X4) or NL4.3-Ada (R5) viruses WT or dU for 48 h, and co-cultured with PBMCs. After 24 h of co-culture, levels of IFN-I released in supernatants were measured. A representative example of absolute levels **(D)** or relative percentages **(E)** of IFN-I detected after co-culture of the indicated infected primary CD4+ T cells with PBMCs (n = 11) are shown. **(F-G)** MT4 cells were mock-infected or infected with GFP-marked NL4.3 virus lacking Vpu (dU) or encoding either NL4.3 Vpu (WT), T/F Suma Vpu (pNL-Suma) or T/F CH077 Vpu (pNL-77). Infected cells were co-cultured with PBMCs 48 h post infection. After 24 h of co-culture, levels of IFN-I released in supernatants were measured. A representative example of absolute levels **(F)** or relative percentages **(G)** of IFN-I detected after co-culture of the indicated infected MT4 donor cells with PBMCs (n = 5) are shown. In all analyses, the amount of IFN-I released by PBMCs in contact with dU HIV-infected cells was set at 100%. Two-tailed paired *t*-test was used in C and E and repeated measures ANOVA with Bonferroni’s multiple comparison test in G (*** p<0.001, ** p<0.01, * p<0.05, ns not significant (p>0.05)). Error bars represent standard deviations (SD).

To examine the role of Vpu during innate sensing of infected cells by PBMCs, MT4 T cells were infected with GFP-marked wild type (WT) or Vpu-deficient (dU) HIV-1 viruses. As previously reported [17,18], Vpu triggered a significant but incomplete downregulation of surface BST2, resulting in an efficient release of HIV-1 particles (S1A–S1D Fig). In parallel, infected MT4 cell populations with a similar percentage of infected cells (GFP-positive cells) were co-cultured for 18–24 h with freshly isolated human PBMCs (Fig 1A). Consistent with previous results [6], IFN-I was very efficiently detected only after co-culture of infected T cells with PBMCs (Fig 1B). Interestingly, levels of IFN-I released in co-cultures were decreased in a Vpu-dependent manner (Fig 1B). Analysis of innate sensing of HIV-1-infected cells by PBMCs from different donors revealed that the presence of Vpu in infected donor cells led to an ~50% reduction in IFN-I production (Fig 1C). We confirmed our results using different Vpu variants as well as primary CD4+ T cells as virus-producing cells. Since pDCs express both HIV co-receptors, CXCR4 and CCR5, primary CD4+ T cells were infected with GFP-marked WT or dU NL4.3 encoding CXCR4 or CCR5-tropic envelopes (NL4.3 and NL4.3-Ada). It is important to note that NL4.3-Ada encodes a Vpu protein from the primary Ada isolate. As observed with MT4 infections, production of IFN-I triggered upon sensing of HIV-infected primary T cells by PBMCs was decreased by ~50% in the presence of Vpu (NL4.3) or Vpu (Ada) (Fig 1D and 1E). Furthermore, similar results were also obtained when NL4.3 virus encoding Vpu variants from transmitter/founder (T/F) strains Suma and CH077 were used. These Vpu variants decreased innate sensing as efficiently as NL4.3 Vpu (Fig 1F and 1G), a finding that was consistent with their comparable ability to downregulate and antagonize BST2 (S1E, S1F and S1G Fig). Collectively, these results suggest that Vpu-mediated BST2 antagonism suppresses the production of IFN-I triggered upon sensing of HIV-1 infected cells by PBMCs independently of co-receptor usage. Additionally, they further indicate that this property is a conserved feature of Vpu variants from primary and T/F virus strains.

### Vpu limits the antiviral response of pDCs upon sensing of HIV-1 infected cells

**Fig 2:**
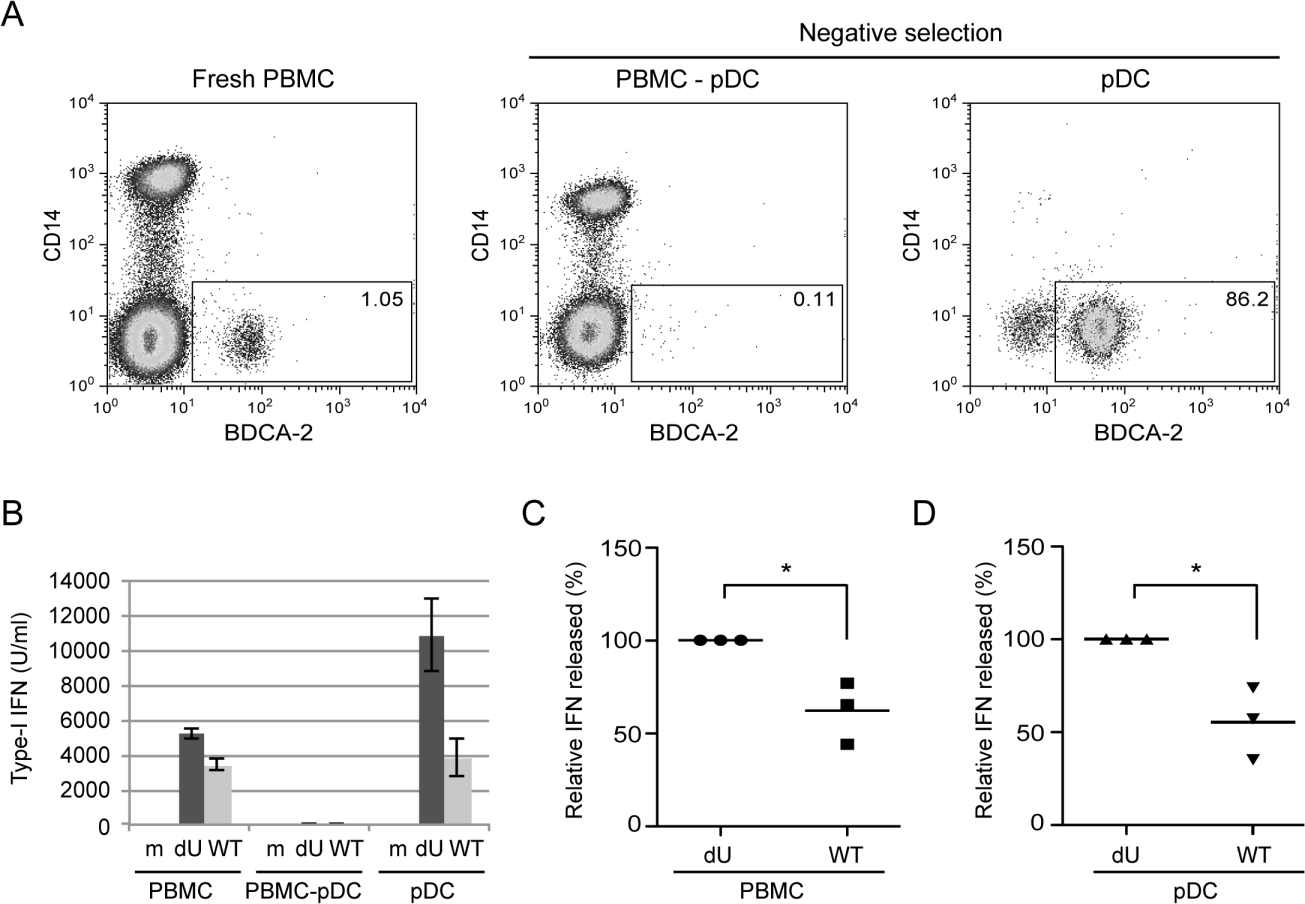
Vpu attenuates IFN-I production upon sensing of HIV-infected cells by pDCs. **(A)** PBMCs, pDC-depleted PBMCs (PBMC-pDC) and enriched pDC populations were stained using anti-CD14 and anti-BDCA-2 Abs and analyzed by flow cytometry. The percentage of pDC cells (BDCA-2+/CD14-) is indicated for each condition. **(B)** A representative example of the absolute levels of IFN-I released in the supernatants of co-cultures of the indicated infected MT4 cells with whole PBMCs, PBMC-pDC, or enriched pDC is shown. **(C-D)** Relative percentages of IFN-I released in supernatants after co-culture of the indicated infected MT4 cells with PBMCs **(C)** or enriched-pDCs **(D)** are shown. The amount of IFN-I released in co-cultures with dU-infected cells was set at 100% (n = 3). Two-tailed paired *t*-test was used (* p<0.05). Error bars represent SD.

Since pDCs are responsible for the vast majority of the IFN-I produced during HIV innate sensing, we then examined whether the differential regulation of IFN-I production mediated by Vpu could be observed in co-cultures with enriched pDCs. To this end, HIV-infected MT4 cells were co-cultured with PBMCs, pDC-depleted PBMCs or enriched populations of pDCs (Fig 2A). In the absence of pDCs, PBMCs lost the ability to sense infected MT4 cells (Fig 2B). In contrast, virus recognition was re-established in the presence of enriched pDC fractions (Fig 2B). Importantly, the differential regulation of IFN-I production mediated by Vpu was observed when enriched pDCs were co-cultured with infected MT4 cells (Fig 2C and 2D). These results indicate that Vpu modulates the antiviral response of pDCs upon sensing of HIV-1 infected cells.

### The canonical mechanism of HIV-1 innate sensing is not altered in the absence of Vpu

**Fig 3:**
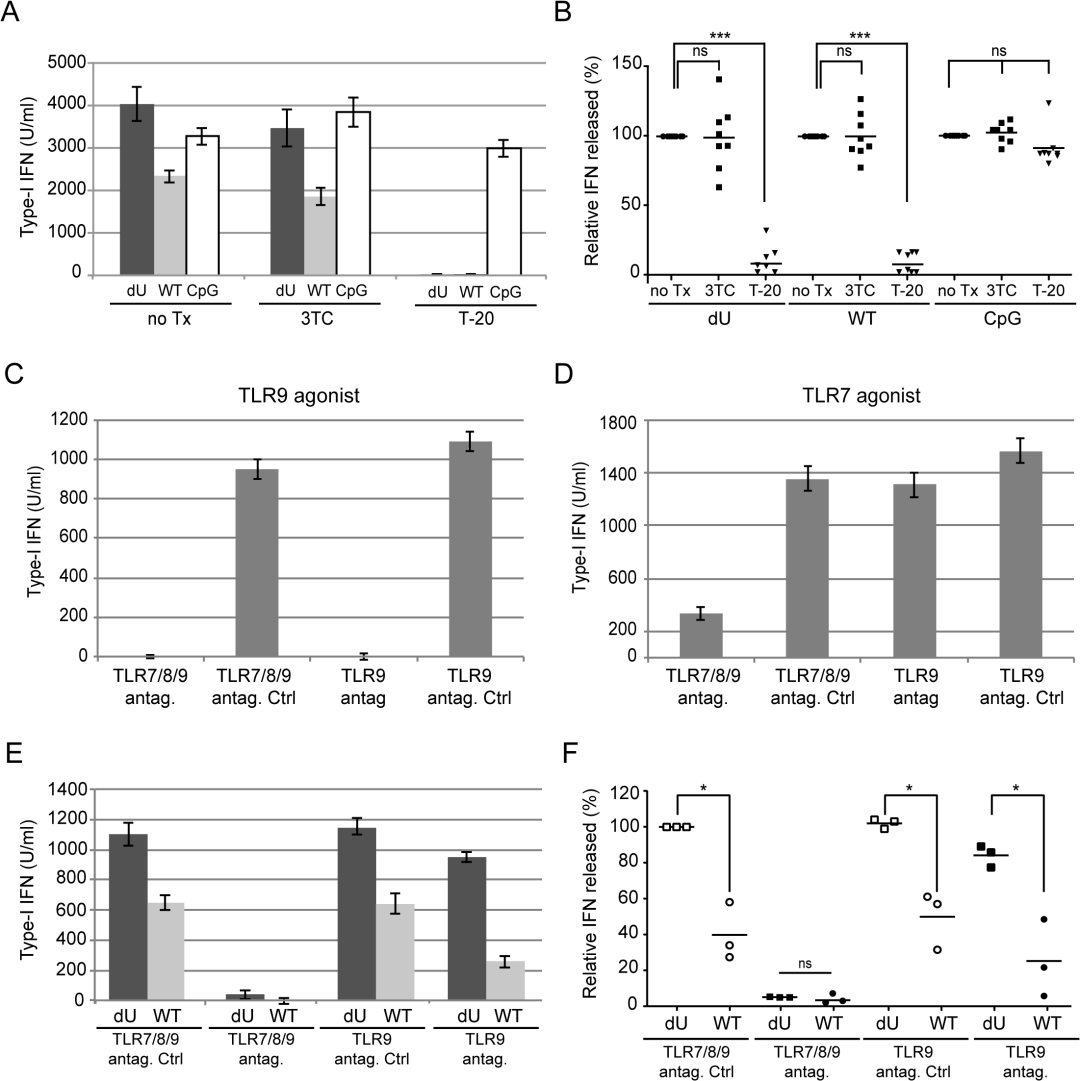
Innate sensing of WT or Vpu-defective HIV-infected T cells requires Env-dependent viral fusion and is largely dependent on TLR7. MT4 cells were mock-infected (m) or infected with GFP-encoding NL4.3 variants (WT or dU) as indicated. **(A-B)** Cells were left un-treated (no Tx) or were treated with T-20 prior to co-culture with PBMCs. To assess the effect of inhibiting reverse transcription, PBMCs were treated with 3TC prior to co-culture with infected MT4 cells. As a positive control, CpG was added to inhibitor-treated or untreated mock-infected cells. A representative example of absolute levels **(A)** or relative percentages **(B)** of IFN-I detected after co-culture of WT or dU HIV-infected MT4 cells with PBMCs in the presence or absence of inhibitors are shown. Results are expressed relative to values obtained in the no-Tx samples (n = 8). **(C-F)** PBMCs were pre-treated with either TLR9 or TLR7/8/9 antagonists (antag.) or their respective controls (antag. Ctrl) prior to TLR agonist treatment **(C-D)** or to co-culture with the indicated infected cells **(E-F)**. A representative example of absolute levels of IFN-I detected after treatment with either TLR9 agonist (CpG-A) **(C)** or TLR7 agonist (R848) **(D)** is shown. A representative example of absolute levels **(E)** or relative percentages **(F)** of IFN-I produced in the indicated co-cultures in the presence of TLR antagonists or controls are shown. The amount of IFN-I released by PBMCs in contact with dU HIV-infected cells in the presence of the TLR7/8/9 antagonist control was set at 100% (n = 3). Two-tailed paired *t*-test was used. (*** p<0.001, * p<0.05, ns not significant (p>0.05)). Error bars represent SD.

Previous studies reported that pDCs recognize WT HIV-infected cells in a large part through TLR7 and by a process that involves Envelope (Env)-dependent transfer of virus across cell contacts [6,8]. Since detection of infected cells by pDCs was enhanced in the absence of Vpu, we examined whether sensing in that context still involved recognition of viral genomic RNA by TLR7. To this end, we first analyzed innate sensing of WT and dU HIV-infected cells in the presence or the absence of inhibitors of either viral fusion or reverse transcription (T-20 and 3TC, respectively). While treatment of HIV-producing cells with T-20 is expected to affect the Env-dependent fusion of transferred ssRNA-containing virions to pDCs, treatment of PBMCs with 3TC will specifically inhibit the conversion of viral RNA into proviral DNA in pDCs. Our results reveal that innate sensing of both WT and dU HIV-infected cells was dependent on viral fusion but not on reverse transcription (Fig 3A and 3B). These findings indicate that Env-dependent fusion of viral particles is required in both contexts and further suggest that viral genomic RNA is likely the primary contributor to WT and dU HIV sensing by pDCs, at least during the first 24 h of the co-culture. Similarly, we tested the effect of TLR9 (ODN_TTAGGG,_ Invivogen) and TLR7/8/9 [27] antagonists (Fig 3C and 3D). These antagonists displayed minimal cytotoxic effects at the concentration that were used. Consistent with the fact that the TLR9 pathway is largely inactivated in pDCs presumably through an interaction of gp120 with BDCA-2 [12,13], we observed only a modest effect of the TLR9 antagonist on pDC response to WT and dU HIV-infected cells (Fig 3E and 3F). In contrast, inhibition of both endosomal TLRs present in pDCs, namely TLR7 and 9 [1] with the TLR7/8/9 antagonist resulted in an almost complete abrogation of IFN-I production in response to both WT and dU HIV-infected cells. These findings support the notion that innate sensing of both WT and dU HIV-1 infected cells relies in a large part on the activation of the TLR7 pathway.

### Vpu-mediated control of IFN-I production by pDCs is dependent on the presence of BST2 at the surface of infected donor cells

**Fig 4:**
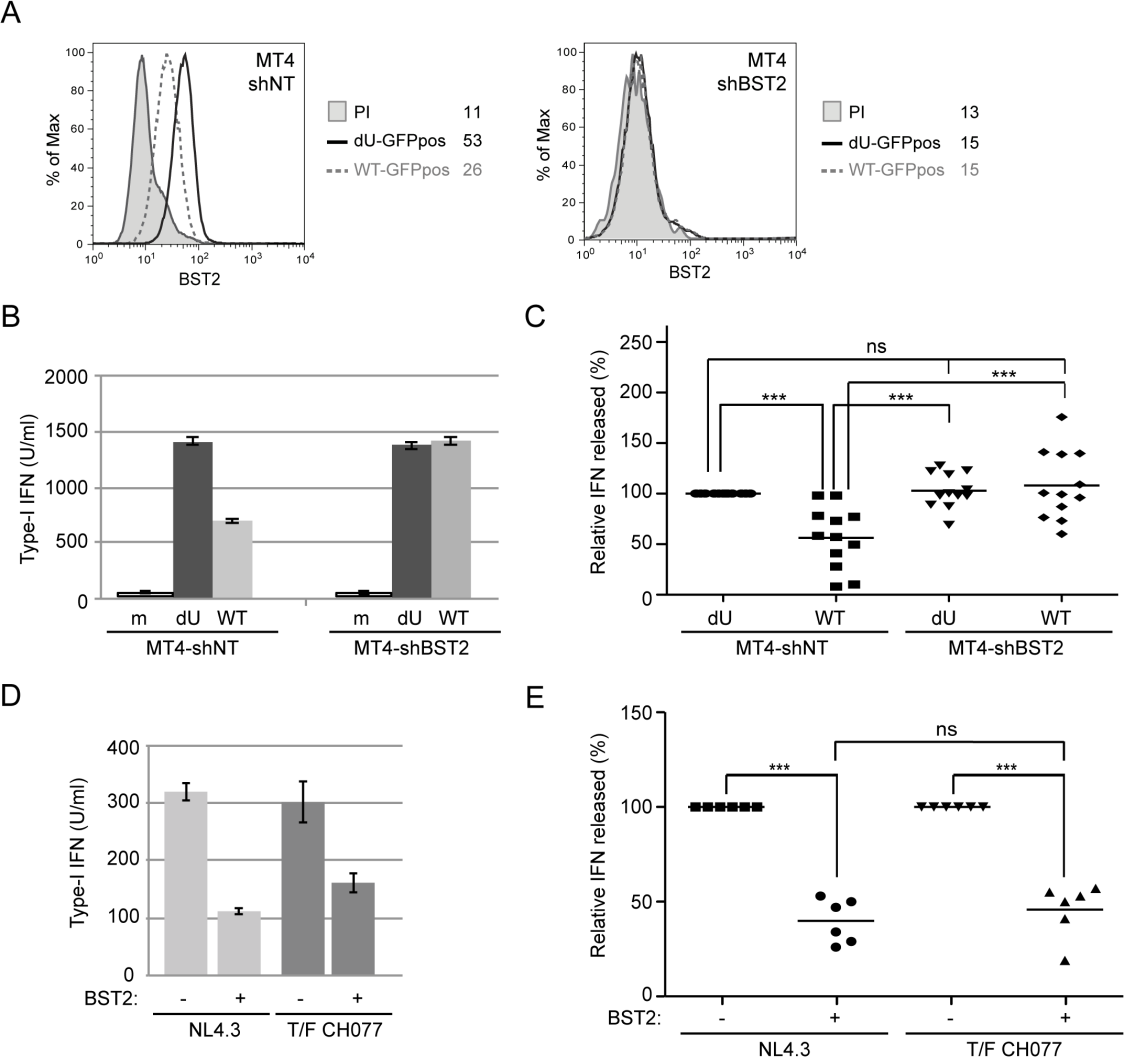
Vpu-mediated control of IFN-I production by pDCs requires the presence of BST2 on infected donor cells. **(A-C)** Control (MT4-shNT) or BST2-depleted (MT4-shBST2) MT4 cells were mock-infected, or infected with GFP-marked NL4.3 WT or dU viruses for 48 h. **(A)** Surface expression of BST2 on GFP-positive MT4 cells infected with WT (dashed grey histogram) or dU (solid black histogram) was evaluated by flow cytometry. Mean fluorescence intensity (MFI) values are indicated for each sample (staining using pre-immune rabbit serum, PI, shaded grey histograms). **(B-C)** The indicated MT4 donor cells were co-cultured with PBMCs. After 24 h, levels of bioactive IFN-I were measured in supernatants. A representative example of absolute levels **(B)** or relative percentages **(C)** of IFN-I produced after co-culture of the indicated infected MT4 cells with PBMCs are shown. The amount of IFN-I released by PBMCs in contact with dU HIV-infected MT4-shNT cells was set at 100% (n = 12). **(D-E)** MT4-shNT (BST2 +) or MT4-shBST2 (BST2 -) cells were infected with GFP-marked NL4.3 WT or T/F CH077 viruses for 48 h. Similar number of p24+ infected cells were then co-cultured with PBMCs. After 24 h, levels of bioactive IFN-I were measured in supernatants. A representative example of absolute levels **(D)** or relative percentages **(E)** of IFN-I produced after co-culture of the indicated infected MT4 cells with PBMCs are shown. The amount of IFN-I released by PBMCs in contact with infected MT4-shBST2 cells was set at 100% (n = 6). Repeated measures ANOVA with Bonferroni’s multiple comparison test was used. (*** p<0.001, ns not significant (p>0.05)). Error bars represent SD.

Having shown that Vpu could dampen IFN-I production during innate sensing of infected cells, we next evaluated the contribution of BST2 towards this process. For this purpose, we generated BST2-depleted (MT4-shBST2) as well as control (MT4-shNT) MT4 cell populations. BST2 downregulation (Fig 4A) and enhanced virus particle release (S2A and S2B Fig) were observed in MT4-shNT cells infected with WT virus when compared to dU virus. In contrast, depletion of BST2 abolished the requirement of Vpu for efficient viral particle release (S2A and S2B Fig). Indeed, the absolute amounts of virus released after WT or dU infections were comparable to those released from WT-infected MT4-shNT cells (S2A Fig). These infected cells were then co-cultured with PBMCs. While the absence of BST2 did not affect the level of IFN-I produced upon sensing of dU HIV-infected cells, it completely abolished the reduction of IFN-I production mediated by Vpu. Indeed, upon depletion of BST2, WT and dU HIV-infected cells were sensed to a degree comparable to that observed with dU HIV-infected MT4-shNT control cells (Fig 4B and 4C). Similar results were observed with several independently depleted cell populations, thus excluding confounding effects resulting from cellular selection. These findings demonstrate that the control of IFN-I production mediated by Vpu is dependent on the presence of BST2 on infected donor cells. It is unlikely that the more efficient IFN-I production observed in co-cultures with Vpu-defective HIV-infected T cells is due to a more effective presentation of dU virions at the cell surface for transmission and/or sensing by pDCs. Indeed, in condition where BST2 was depleted, dU HIV-infected MT4 cells were sensed as efficiently as dU HIV-infected control cells (Fig 4B and 4C), despite the fact that virus release efficiency was clearly different in these two conditions (S2A and S2B Fig). Using these cell lines, we further showed that the X4/R5-tropic CH077 T/F virus was as efficient as the NL4.3 virus at suppressing innate sensing in the presence of BST2 (Fig 4D and 4E), thus providing additional evidence that T/F virus have the capacity to control IFN-I production by pDCs. To further confirm the role of BST2 in the Vpu-mediated control of innate sensing, we tested in our co-culture system a virus encoding a TM domain mutant of Vpu (Vpu A_10-14-18_L) that is drastically attenuated in its ability to bind and antagonize BST2 [28] (S2C and S2D Fig). This mutant virus was found to be phenotypically identical to dU virus during innate sensing (S2E and S2F Fig), further supporting the crucial role that Vpu-mediated BST2 antagonism plays in regulating pDC antiviral responses.

**Fig 5:**
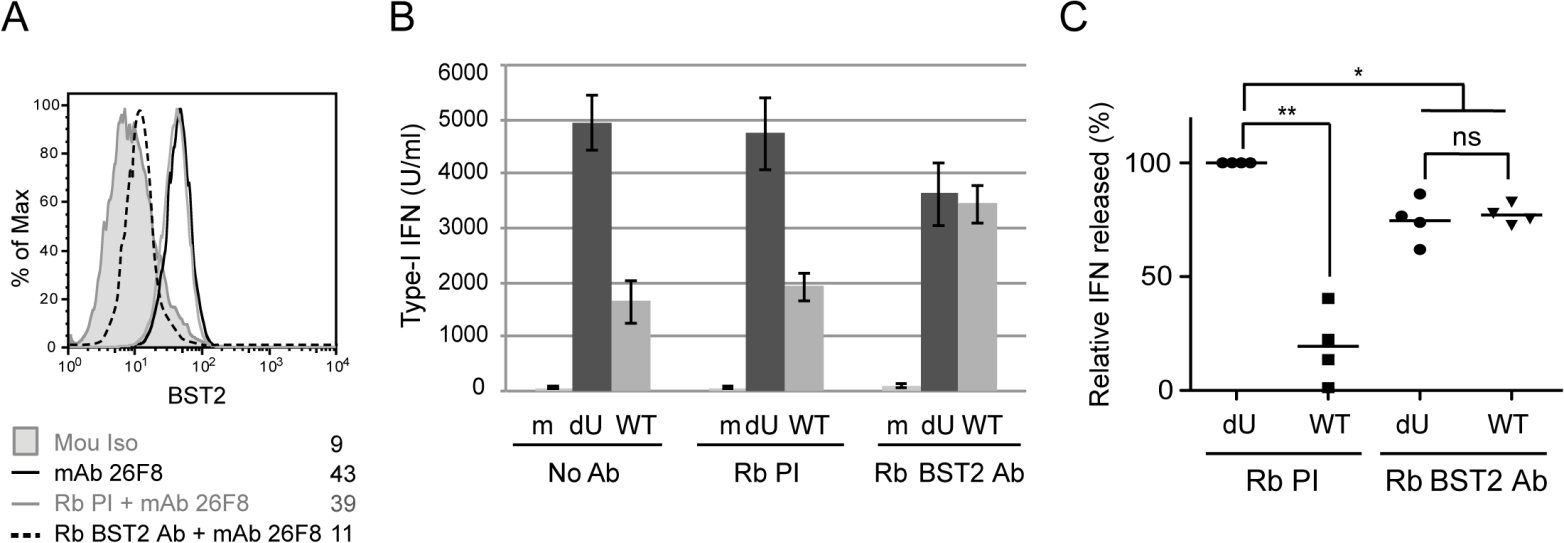
BST2 at the surface of infected cells is required for Vpu-mediated control of IFN-I production. MT4 cells were mock-infected or infected with GFP-marked NL4.3 WT or dU viruses and pre-incubated with anti-BST2 rabbit polyclonal (Rb BST2 Ab) or pre-immune (Rb PI) Abs or left untreated (No Ab). **(A)** Mock cells were subsequently stained for surface BST2 using mAb 26F8 and analyzed by flow cytometry. As a positive control, cells were directly stained with mAb 26F8. **(B-C)** The indicated MT4 cells were co-cultured with PBMCs. After 24 h, levels of IFN-I released in supernatants were measured. A representative example of absolute levels **(B)** or relative percentages **(C)** of IFN-I produced after co-culture of PBMCs with infected MT4 cells pre-treated with the indicated Abs are shown. The amount of IFN-I released by PBMCs in contact with dU HIV-infected cells in presence of Rb PI was set at 100% (n = 4). Two-tailed paired *t*-test was used (** p<0.01, * p<0.05, ns not significant (p>0.05)). Error bars represent SD.

We next assessed whether Vpu-mediated reduction of IFN-I production was dependent on the presence of accessible BST2 molecules at the surface of infected donor cells. To this end, infected MT4 cells were pre-incubated with BST2-specific polyclonal rabbit antibodies (Rb BST2 Ab) directed against the extracellular domain of the molecule or rabbit pre-immune serum (Rb PI). Miyagi and colleagues previously reported that binding of similar anti-BST2 polyclonal Abs to BST2 did not trigger internalization of the protein but rather stabilized the restriction factor on the cell surface [29]. A saturation of potentially available cell-surface BST2 with the BST2-specific polyclonal Rb Ab was confirmed by the near absence of BST2 staining detected with an anti-BST2 mouse monoclonal antibody (mAbs 26F8) 18 h after addition of Rb BST2 Abs (Fig 5A). Hence, mock or infected MT4 cells were pre-incubated with BST2-specific polyclonal Rb Abs or the Rb PI control prior to the 18 h co-culture with PBMCs. Fig 5B and 5C reveals that treatment with Rb BST2 Abs abolished the differential innate sensing observed between WT and dU HIV infected cells. Noticeably, in both cases the levels of IFN-I were lower than those detected in dU HIV infected cells treated with control Abs, suggesting a potential slight interfering effect of the rabbit polyclonal anti-BST2 serum on pDCs. These results combined with those obtained upon depletion of BST2 suggest that control of IFN-I production by Vpu requires that BST2 be expressed at the surface of infected donor cells and available for potential interactions.

### Residual BST2 molecules are detected outside of virus assembly sites in the presence of Vpu

**Fig 6:**
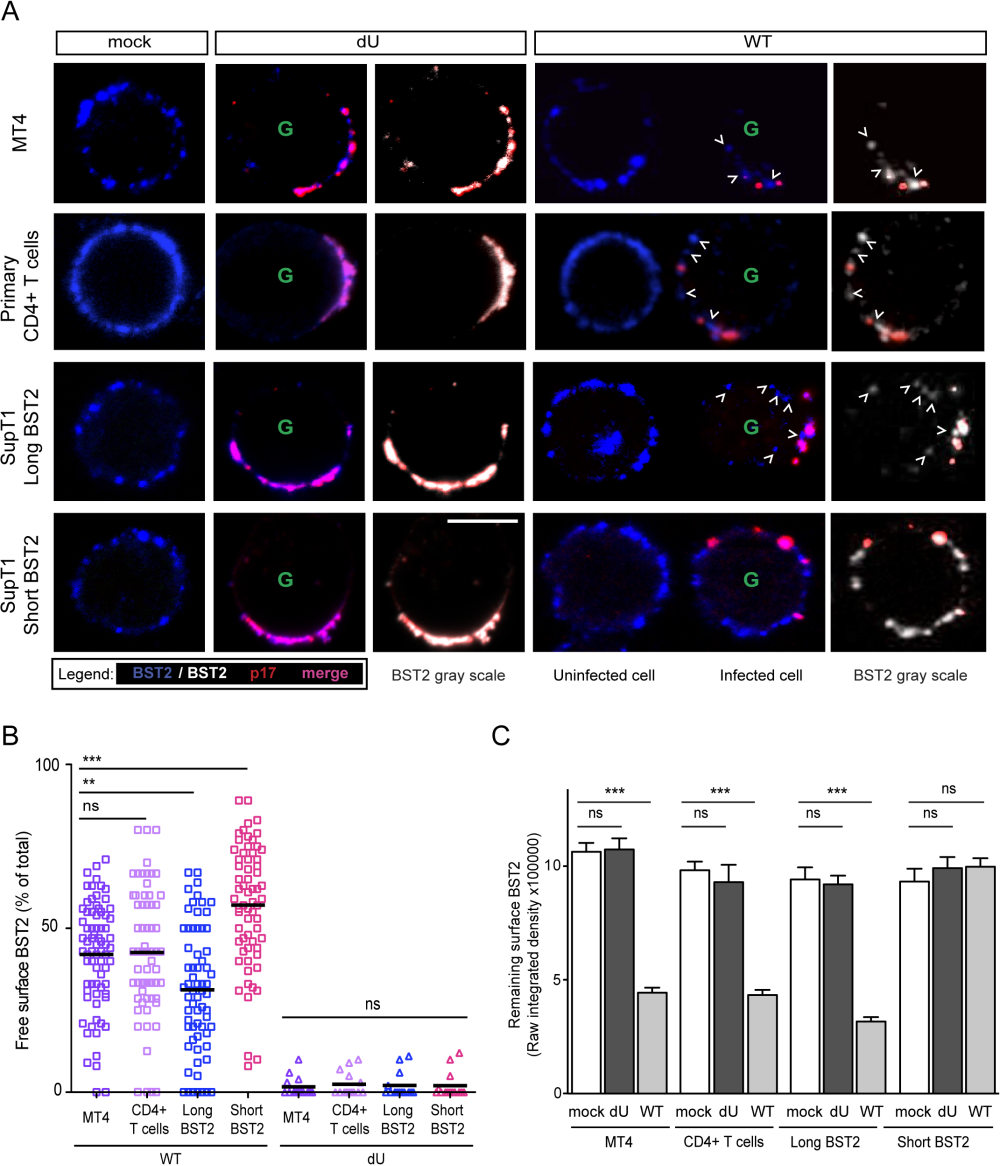
Residual BST2 clusters are detected outside virus assembly sites in the presence of Vpu. MT4, primary CD4+ T cells, SupT1-shortBST2 and SupT1-longBST2 cells were mock-infected (mock) or infected with VSV-G-pseudotyped NL4.3-Ada-GFP WT or dU viruses. **(A)** Cells were stained with anti-BST2 Abs (blue), fixed, permeabilized and then sequentially stained with anti-p17 Abs (red). Infected cells (GFP+) are marked with a green letter G. An uninfected cell is shown next to WT-infected cells as indicated. Clusters of free BST2 are marked with white open arrows. White bar = 10 μm. **(B)** The number of residual BST2 clusters not co-localizing with p17 (designated as free BST2) per cell was calculated and expressed as the percentage of the total number of surface BST2 clusters. **(C)** Quantitative analysis of surface BST2 was determined as described in Materials and Methods. One way ANOVA with Bonferroni’s multiple comparison test was used (*** p<0.001, ** p<0.01, ns not significant (p>0.05)). Error bars indicate the standard error of the mean after analysis of at least 50 distinct cells.

One possible model to explain the efficient IFN-I production by pDCs during sensing of dU HIV-infected cells could be that tethered viral clusters at the surface of infected cells would occlude BST2 from potential interaction with the ILT7 inhibitory receptor on pDCs. In contrast, in cells infected with WT HIV, Vpu would downregulate BST2 sufficiently to prevent restriction of virion release but maintain enough BST2 to engage and activate ILT7. Indeed, it was recently reported that besides inducing a downregulation of BST2, Vpu has the ability to displace the restriction factor from sites of viral assembly as a means to counteract BST2 restriction [30,31]. To assess the frequency of surface BST2 molecules that are not potentially engaged in restriction of progeny virions, we performed co-localization studies of BST2 and HIV Gag-p17, a marker of assembling HIV, on GFP-positive infected MT4 and primary CD4+ T cells. As previously reported [30], surface BST2 accumulated in patches that perfectly co-localized with p17 in the absence of Vpu (Figs 6A, top panels and S3E). BST2 molecules localizing outside of viral assembling sites (we refer to this pool as ˝free BST2˝) were rarely found (Fig 6B), suggesting that in dU HIV-infected cells the majority of surface BST2 appears engaged in restricting assembling viruses. Consistent with the flow cytometry data of S1C and S2D Fig and our previous observations [32], surface BST2 levels were significantly decreased in the presence of Vpu and this was linked to a reduction in p17 accumulation at the cell periphery (Figs 6A, 6C and S3E) in agreement with efficient virus particle release (S1A and S1B Fig). Noticeably, in this context, we could detect clusters of BST2 that were not co-localizing with p17 (marked with white open arrows) (Fig 6A and 6B), suggesting that Vpu displaces a residual pool of surface BST2 from virus assembly sites.

Two isoforms of human BST2 derived from alternative translation initiation from highly conserved methionine residues in the cytosolic domain were identified in both immortalized cell lines and primary cells [33]. Both isoforms are able to restrict virion release. The short isoform, which lacks 12 N-terminal residues, including conserved tyrosine and serine-threonine motifs present in the long isoform, was reported to be significantly more resistant to Vpu-mediated degradation, surface downregulation and antagonism as compared to the long isoform [33,34]. To assess the distribution of these BST2 isoforms relative to assembling virions in the presence or absence of Vpu, SupT1 T cells, which naturally do not express BST2 [17], were supplemented with either the long Vpu-sensitive BST2 isoform or the short BST2 isoform (S3A and S3B Fig). In the absence of Vpu, both isoforms were found to similarly co-localize with p17 (Fig 6A bottom panels), consistent with their comparable ability to restrict virus particle release (S3C and S3D Fig). Vpu induced a significant depletion of the long BST2 isoform at the cell surface, whereas it had only minor effects on short BST2 isoform surface levels (Figs 6A, 6C and S3B). Despite the lack of short isoform downregulation by Vpu, we did not observe a marked accumulation of p17 staining at the periphery of T cells (Fig 6A), further confirming that Vpu-mediated BST2 antagonism can occur in the absence of BST2 downregulation (S3B, S3C and S3D Fig). This was in sharp contrast to dU HIV-infected SupT1-shortBST2 cells which expressed similar levels of surface BST2 but displayed a noticeable accumulation of cell-associated p17 (Figs 6A, 6C, S3B, S3C and S3D). Interestingly, in the context of Vpu-expressing infected cells, significant amounts of short BST2 molecules (both in terms of frequency and intensity) could be detected outside viral assembly sites (Fig 6), indicating that a pool of short BST2 isoform molecules is potentially accessible for interactions in the presence of Vpu. Nevertheless, it is important to point out that a significant pool of free BST2 could also be detected in SupT1 cells expressing the long BST2 isoform in the presence of Vpu, albeit their levels (as indicated by integrated pixel density) and frequency outside viral assembly sites were lower as compared to the short isoform (Fig 6). Importantly, in none of the tested cell lines nor in primary CD4+ T cells, were we able to detect significant amounts of free BST2 in the absence of Vpu (Fig 6B), even though surface BST2 levels remained comparable to those of mock-infected cells (Fig 6A and 6C). These observations were confirmed using the gp120 Env protein as an additional marker of viral assembly sites, as previously described [35]. Co-localization studies of BST2 and Env in the presence or absence of Vpu further documented that Vpu induced a redistribution of BST2 outside assembly sites in primary CD4+ T cells and in SupT1 cells expressing the short BST2 isoform (S3F Fig). Overall, our data indicate that in the absence of Vpu, the pool of free BST2 at the surface of infected cells is limited. In contrast, in the presence of Vpu, there is a residual pool of surface BST2 that is excluded from viral budding sites and thus potentially accessible for interaction with ILT7 on pDCs. We expect this pool of BST2 to be predominantly composed of the short isoform.

### A BST2 mutant that is unable to entrap HIV-1 particles represses IFN-I production by PBMCs independently of Vpu

**Fig 7:**
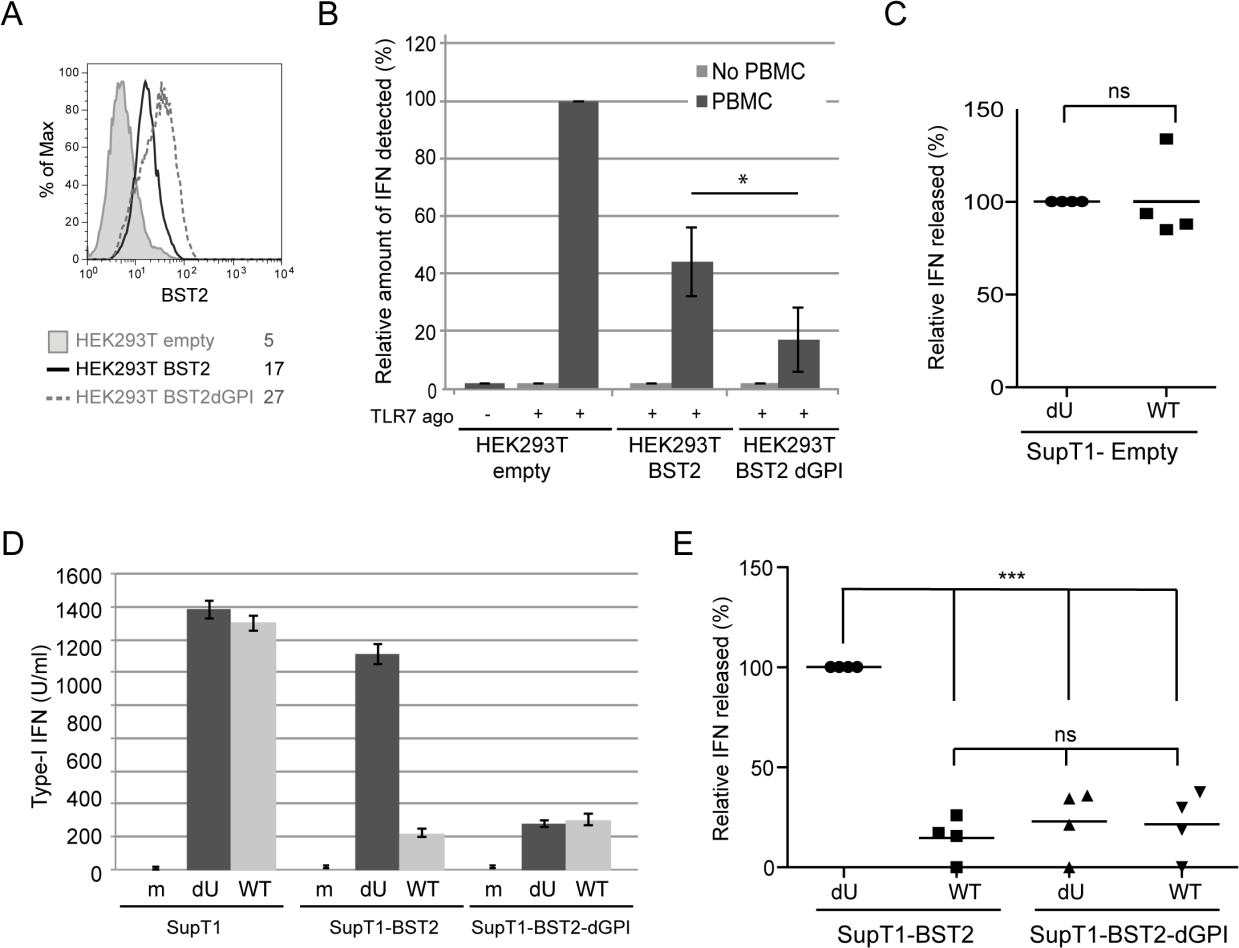
Effect of a BST2 GPI anchor mutant on Vpu-mediated control of IFN-I production by pDCs. **(A-B)** HEK293T cells were transfected with either an empty plasmid or plasmids expressing BST2 or BST2-dGPI 48 h prior to co-culture with PBMCs. **(A)** Surface expression of BST2 was evaluated 48 h post transfection by flow cytometry in controls cells (shaded grey histogram), as well as in cell expressing BST2 (solid black histogram), or BST2-dGPI (dashed grey histogram). Mean fluorescence intensity (MFI) values are indicated for each sample **(B)** After 6 h of co-culture, samples were untreated or treated with Imiquimod (TLR7 agonist) and levels of bioactive IFN-I in supernatants were measured 18 h later. The amount of IFN-I released by PBMCs in contact with HEK293T cells transfected with the empty plasmid in presence of the TLR 7 agonist was set at 100% (n = 4). As a control, transfected HEK293T cells were treated with TLR7 agonist without PBMCs. **(C)** Percentage of IFN-I released after co-culture of infected SupT1-Empty with PBMCs normalized to the value obtained with dU HIV-infected SuptT1 cells (100%) (n = 4). **(D)** A representative example of absolute levels of IFN-I produced after co-culture of mock or infected-SupT1,-SupT1-BST2 or-SupT1-BST2-dGPI cells with PBMCs is shown. **(E)** Relative percentages of IFN-I produced after co-culture of infected-SupT1-BST2 or SupT1-BST2-dGPI cells with PBMCs are shown. The amount of IFN-I released by PBMCs in contact with dU HIV-infected SupT1-BST2 cells was set at 100% (n = 4). Two-tailed paired *t*-test was used in B and C (* p<0.05, ns not significant (p>0.05)). Repeated measures ANOVA with Bonferroni’s multiple comparison test was used in E (*** p<0.001, ns not significant (p>0.05)). Error bars represent SD.

The BST2 GPI anchor is required for BST2-mediated entrapment of virions and in its absence HIV particles are no longer retained at the surface of infected cells, as assessed by their efficient release [17]. The ability of the BST2-dGPI mutant to block the TLR7 pathway was tested. HEK293T were transfected with plasmids expressing either WT BST2 or a BST2-dGPI mutant and the levels of BST2 was evaluated by flow cytometry (Fig 7A). When PBMCs were co-cultured with BST2-expressing HEK293T cells in the presence of a TLR7 agonist, IFN-I production was reduced by ~66% as compared to control (Fig 7B). Importantly, HEK293T cells expressing the BST2-dGPI mutant efficiently repressed IFN-I production as well, highlighting the fact that the BST2-dGPI anchor is not required for inhibition of TLR7-mediated IFN-I production by BST2 (Fig 7B). Of note, no IFN-I was detected after co-culture of PBMCs with HEK293T cells in the absence of TLR7 agonist (Fig 7B). Sup T1 control cells (Empty) as well as SupT1 cells expressing BST2-dGPI at levels comparable to those detected in SupT1-WT BST2 cells were infected with WT or dU viruses prior to co-culture with PBMCs (S4A Fig). These cells exhibited the expected phenotype in terms of virus release (S4B and S4C Fig) and surface BST2 modulation by Vpu (S4A Fig). Consistent with the results obtained with BST2-depleted MT4 cells (Fig 4), BST2-deficient SupT1 cells infected with WT or dU virus triggered similar levels of IFN-I upon their co-culture with PBMCs (Fig 7C). In contrast, in co-cultures with BST2-expressing SupT1 cells, HIV-1 WT triggered significantly less IFN-I than its dU virus counterpart (Fig 7D and 7E). Interestingly, expression of BST2-dGPI at the surface of HIV-producing cells very efficiently repressed IFN-I production by PBMCs independently of the presence of Vpu. Indeed, in this context, the extent of repression of IFN-I production was comparable to that observed when PBMCs were co-cultured with WT HIV-infected SupT1-BST2 cells (Fig 7D and 7E). Taking together, these results suggest that trapping of progeny virions by BST2 prevents the restriction factor from eliciting an inhibition of IFN-I production by pDCs.

### Vpu-mediated suppression of IFN-I production requires engagement and activation of the ILT7 pDC receptor by BST2

**Fig 8:**
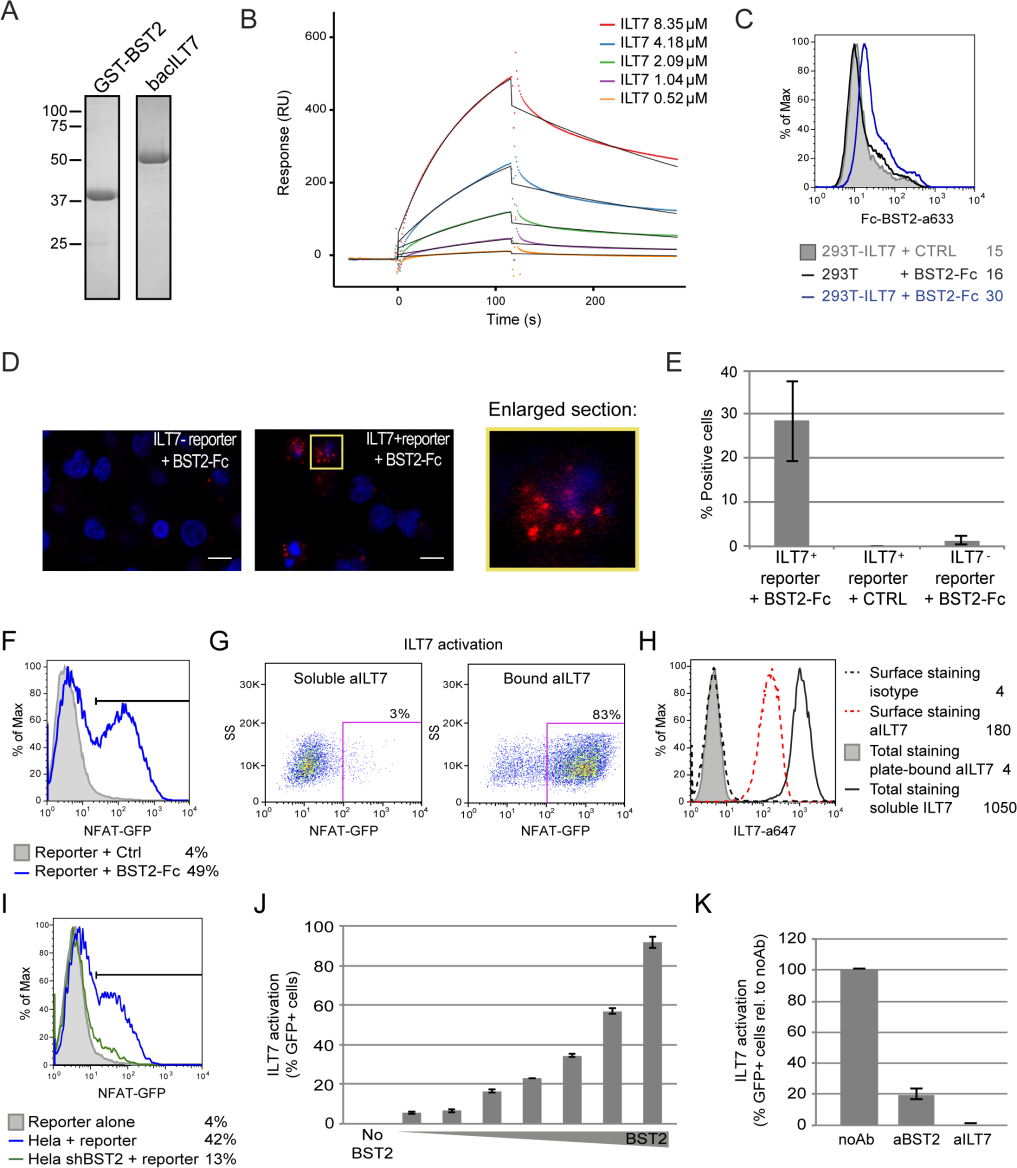
BST2 binds and effectively activates ILT7. **(A-E)** BST2 binds ILT7. **(A)** Purified GST-BST2 and bacILT7 were analyzed by SDS-PAGE and visualized by Coomassie brilliant blue staining. **(B)** Recombinant GST-BST2 pre-coated on the surface of Biacore sensor chips, was mixed with the indicated concentrations of bacILT7. The kinetic response data after subtracting the value from a reference cell coated with GST alone are shown. Kinetic constants (K_D_ = 2.33 μM, k_on_ = 1.25×10^3^ M^-1^s^-1^, k_off_ = 3.08×10^−3^ s^-1^) were derived by fitting the data (dotted lines) to a 1:1 Langmuir model (black lines) using local R_max_ parameters (chi^2^ = 14). **(C)** Control (293T) or ILT7-expressing HEK 293T cells (293T-ILT7) were incubated with control supernatant (CTRL) or with BST2-Fc-containing supernatant (BST2-Fc) prior to crosslinking with DTSSP. Cells were then stained for surface BST2-Fc and analyzed by flow cytometry. **(D-E)** ILT7+ or ILT7- NFAT-GFP reporter cells were incubated with control supernatant (CTRL) or with BST2-Fc-containing supernatant (BST2-Fc). Proximity ligation assay (PLA) was performed using mouse ILT7 mAb and rabbit polyclonal anti-BST2 Abs. A fluorochrome-labeled probe (red) was used to reveal locations of close proximity, and nuclei were highlighted with DAPI staining (blue). **(D)** Images were acquired by confocal microscopy using a 63Å~ objective. Images shown are representative of multiple fields. A magnification of the section marked in yellow is shown beside the panel. White bar = 10 μm. **(E)** The percentage of cells with PLA red staining (% positive cells) was calculated from at least 50 cells per condition. **(F-K)** BST2 effectively activates ILT7. **(F)** ILT7+ NFAT-GFP reporter cells were cultured in the presence or absence of plate-bound BST2-Fc for 24 h and analyzed for GFP expression using flow cytometry. **(G-H)** ILT7+ NFAT-GFP reporter cells were cultured in the presence of plate-bound or soluble anti-ILT7_alexa647 Abs (grey shaded or solid black histograms, respectively) or soluble isotype_alexa647 Ab as negative control (dotted lines). Twenty-four hours later, cells were harvested and samples in contact with the isotype Ab were stained for surface ILT7 only, using the above mentioned Abs for 30 min at 4°C (isotype_alexa647: dotted black histogram and aILT7_alexa647: dotted red histogram). All sample were analyzed by flow cytometry to detect **(G)** the percentage of GFP positive cells and **(H)** anti-ILT7 Abs (surface or total: surface + internalized). **(I-K)** ILT7+ NFAT-GFP reporter cells were co-cultured for 24 h with **(I)** control Hela or BST2-depleted Hela (Hela shBST2) cells or **(J-K)** HEK293T cells expressing either **(J)** increasing amounts of BST2 or **(K)** a fixed amount of BST2 and analyzed by flow cytometry (n = 2). **(K)** Prior to co-cultures, HEK293T-BST2 cells were incubated with rabbit anti-BST2 Abs (aBST2) or ILT7+ NFAT-GFP reporter cells were incubated with anti-ILT7 Abs (aILT7) for 1h or cells were left untreated as control (noAb). Relative percentage of ILT7 activation was plotted as % of GFP+ cells in each condition relative to the no Ab condition, which was set at 100%. Error bars represent SD.

Because surface BST2 was a critical mediator of Vpu-mediated innate immune modulation, we next examined whether the BST2 binding partner, ILT7, expressed on pDCs could be involved in this process as well. The reported interaction between BST2 and ILT7 [25] was confirmed *in vitro* by surface plasmon resonance (K_D_ = 2.33 μM) using recombinant GST-tagged ectodomain of BST2 (GST-BST2, a soluble form containing a region common to the short and the long BST2 isoforms) and baculovirus-expressed soluble ILT7 (Fig 8A and 8B). This interaction was further demonstrated *in cellulo* by flow cytometry and by proximity ligation assay using Fc-tagged ectodomain of BST2 (BST2-Fc) and ILT7-expressing HEK293T cells or a ILT7+ NFAT-GFP reporter cell line [24], respectively (Fig 8C, 8D and 8E). Interestingly, ILT7 activation could be triggered in the reporter cell line using plate-bound BST2-Fc or plate-bound anti-ILT7 Abs but not with soluble anti-ILT7 Abs (Fig 8F and 8G), most likely because of rapid internalization of the receptor-Ab complexes (Fig 8H). Activation of ILT7 could also be detected by co-culture with BST2-expressing cells (Fig 8I and 8J). Importantly, ILT7 activation in the presence of BST2-expressing HEK 293T cells could be blocked when either anti-ILT7 or anti-BST2 Abs were added to the co-culture (Fig 8K). These results indeed confirm that the interaction between these two proteins is capable of triggering ILT7 activation, a signaling event that leads to repression of IFN-I production in pDCs [24,25].

**Fig 9:**
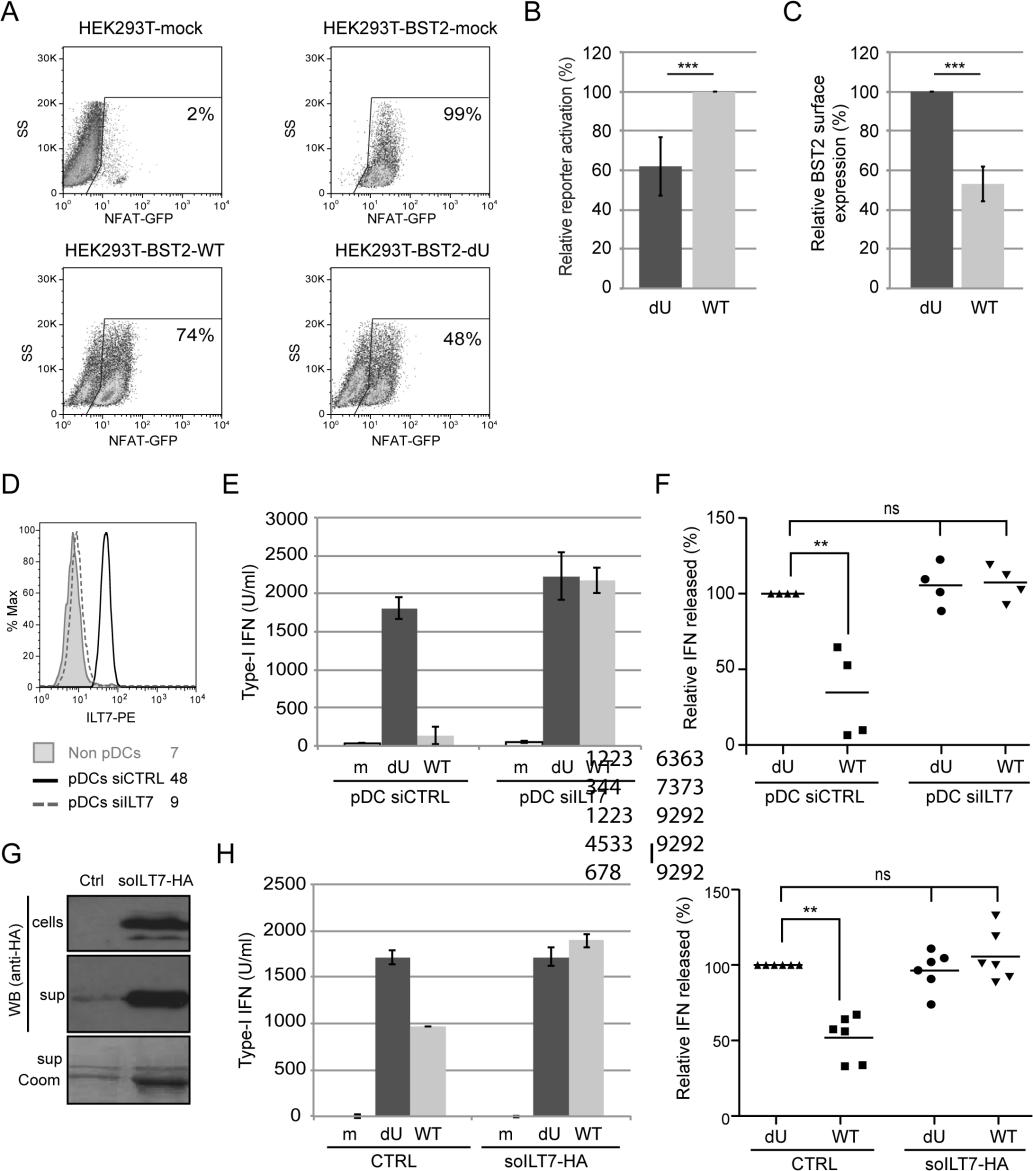
Vpu-mediated control of IFN-I production by pDCs involves engagement and activation of ILT7 by BST2. **(A-C)** Vpu-mediated BST2 antagonism enhances activation of ILT7. ILT7+ NFAT-GFP reporter cells were co-cultured with HEK293T (mock) or BST2-expressing HEK293T cells mock-transfected or transfected with the indicated pNL4.3 constructs (WT or dU). **(A)** Representative example of ILT7 activation as determined by the percentage of NFAT-GFP positive cells measured by flow cytometry. **(B)** Percentage of ILT7 activation after co-culture with the indicated HIV/BST2 expressing HEK 293T cells relative to WT HIV-producing cells (100%) (n = 4). **(C)** Percentage of BST2 surface expression in HEK293T cells after co-transfection of BST2 with the indicated HIV provirus relative to dU HIV-producing cells (100%) (n = 4). **(D-F)** Effect of ILT7 depletion on IFN-I production by pDCs. **(D)** Non-pDC fraction (BDCA-2-) and siRNA-treated enriched pDCs (CD14-/BDCA-2+) were stained using anti-ILT7 Abs as indicated. A representative example of absolute levels **(E)** or relative percentages **(F)** of IFN-I produced after co-culture of control pDCs (pDC-siCTRL) or ILT7-depleted pDCs (pDC-siILT7) with the indicated infected MT4 cells are shown. The amount of IFN-I released by pDC siCTRL in contact with dU HIV-infected cells was set at 100% (n = 4). **(G-I)** Effect of recombinant soluble ILT7 on IFN-I production by pDCs. **(G)** Expression of a HA-tagged soluble ILT7 (soILT7-HA) in HEK 293T cells. Cells and supernatants (sup) were analyzed by Western blot (WB) using anti-HA Abs. Purity of secreted soILT7-HA was confirmed by Coomassie staining (sup Coom). A representative example of **(H)** absolute levels or **(I)** relative percentages of IFN-I production after co-culture of PBMCs with the indicated infected MT4 cells pre-treated with control (CTRL) or soILT7-HA-containing supernatants are shown. The amount of IFN-I released by PBMCs in contact with dU HIV-infected cells in presence of CTRL supernatant was set at 100% (n = 6). Two-tailed paired *t*-test was used in B-C while repeated measures ANOVA with Bonferroni’s multiple comparison test was used in F and I (*** p<0.001, ** p<0.01, ns not significant (p>0.05)). Error bars represent SD.

To examine whether the presence of Vpu in HIV-producing cells could affect ILT7 activation through BST2, we co-cultured BST2-expressing HEK 293T cells producing either WT or dU HIV with ILT7+ NFAT-GFP reporter cells. While ILT7 activation was very effective in co-culture with BST2-expressing cells, it was significantly reduced when these cells were co-expressing HIV (99% vs 74%) (Fig 9A). Noticeably, WT HIV-producing cells were found to significantly enhance ILT7 activation compared to HEK293T cells expressing dU HIV (74% vs 48%) (Fig 9A and 9B). While these latter cells express higher levels of surface BST2, it is expected that a large proportion of these molecules would be restricting progeny viruses (Fig 9C). These results suggest that Vpu-mediated BST2 antagonism allows the presence of a pool of surface BST2 molecules that are capable of engaging and activating ILT7 upon cell-to-cell contact.

To further examine the role of the ILT7-BST2 regulatory axis, we used a specific type of siRNA (see Materials and Methods) to deplete the endogenous ILT7 from enriched pDC cultures without inducing spontaneous IFN-I production nor cell cytoxicity (Fig 9D and 9E). siRNA-treated pDCs were then co-cultured with infected MT4 cells. While control pDCs were found to secrete reduced amounts of IFN-I upon co-culture with WT HIV-infected MT4 cells relative to co-cultures containing dU HIV-infected MT4 cells, depletion of ILT7 in pDCs abrogated such differences (Fig 9E and 9F). Moreover, addition of recombinant soluble ILT7 (soILT7-HA, Fig 9G) to co-cultures of PBMCs and infected MT4 cells similarly abolished the differential IFN-I production detected with WT and dU HIV-infected cells (Fig 9H and 9I). These results demonstrate that Vpu-mediated suppression of IFN-I production is dependent on the expression of ILT7 on pDCs.

## Discussion

**Fig 10:**
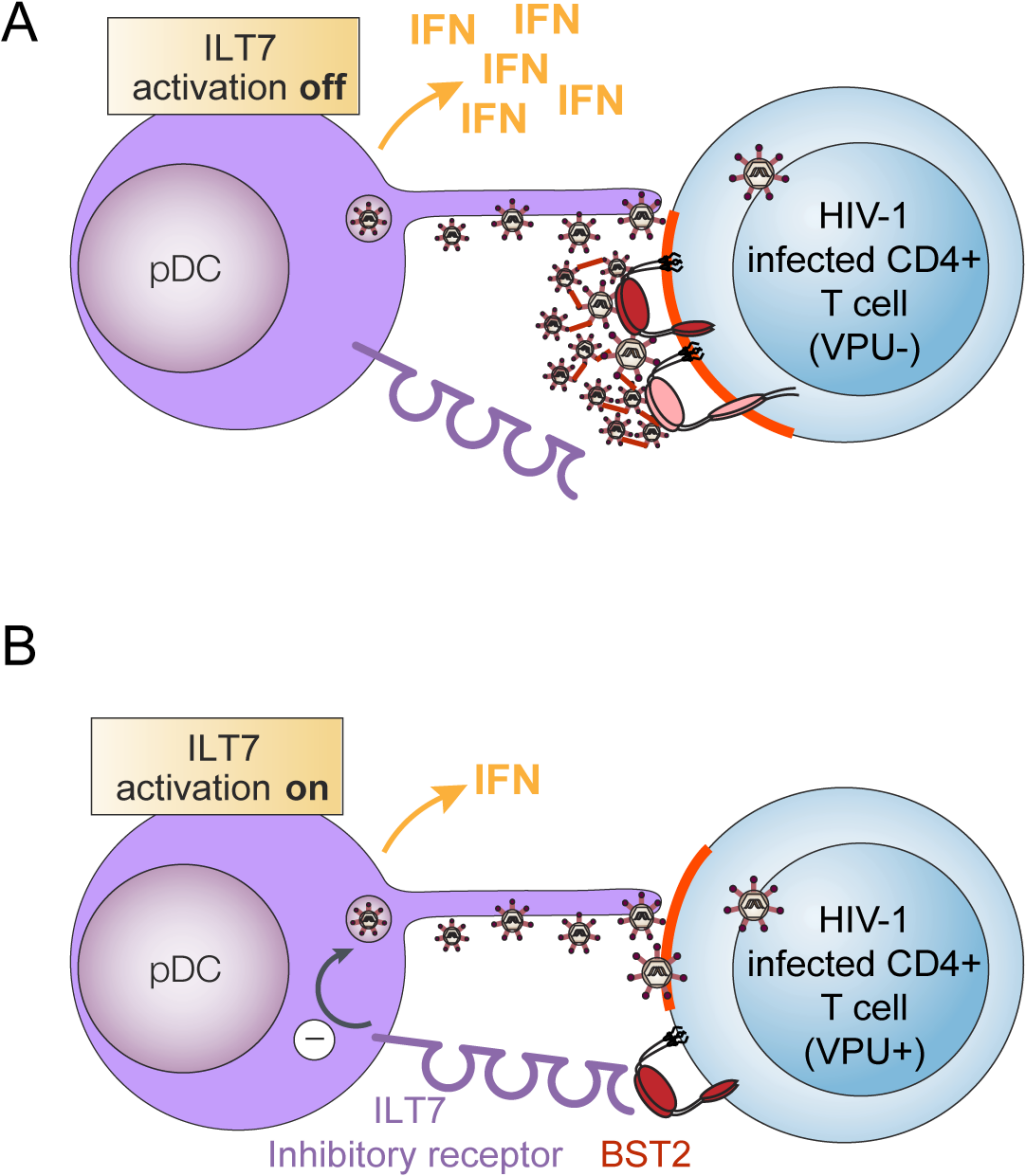
Presence of Vpu in HIV-infected cells limits pDC antiviral responses upon sensing of infected cells. pDCs produce type-I interferon (IFN) upon sensing of HIV-infected cells. This process is negatively regulated when BST2 expressed on HIV producing donor cells interacts with ILT7, a pDC inhibitory receptor. **(A)** In infected cells lacking Vpu, BST2 restricts HIV-1 release by trapping nascent virions at the cell surface. This in turn, obstructs BST2 from interacting with ILT7. In this context, sensing of HIV-infected cells by pDCs will result in efficient IFN-1 production. **(B)** Vpu promotes efficient HIV-1 release in part by down-modulating surface BST2 but also by mislocalizing remaining molecules outside viral assembly sites (highlighted in red). Upon encountering CD4+ T cells infected with WT HIV (Vpu+), pDCs produce reduced amounts of IFN-I because residual surface BST2 molecules are free to interact with ILT7 and activate an inhibitory signal in pDCs.

The Vpu accessory protein has been shown to downregulate many host proteins involved directly or indirectly with anti-HIV immune responses, including among others CD4, NTB-A, CD1d and BST2 [36]. With respect to BST2, it was recently reported that this Vpu-regulated host factor could act not only as a potent inhibitor of HIV release but also as an innate sensor capable of inducing NFκB-dependent proinflammatory responses upon retroviral retention and activation of a Syk-dependent HemITAM in BST2 [37,38]. In the present study, we shed light on a novel Vpu-BST2 interaction, which allows HIV to suppress IFN-I production by pDCs via the negative signaling exerted by the ILT7-BST2 pair (Fig 10).

Using a system of co-culture between HIV-infected T cells and PBMCs or enriched populations of pDCs, we provide evidence that Vpu-mediated BST2 antagonism suppresses the production of IFN-I during innate sensing of HIV-infected T cells. This result initially obtained with a laboratory-adapted Vpu (NL4.3), was further confirmed using Vpu variants from a primary isolate (Ada) and from T/F strains (Fig 1). While this finding could suggest that the increased IFN-I response observed in the absence of Vpu may result from a more effective presentation of BST2-restricted dU HIV-1 virions at the cell surface for transmission or/and sensing by pDCs, a condition counteracted by Vpu, the data herein is not consistent with such a possibility. First, depletion of BST2 in MT4 T cells (Fig 4), a process that abolishes the restriction on HIV-1 release and its dependence on Vpu, did not reduce IFN-I production during innate sensing of dU HIV infected cells but rather enhanced pDC antiviral responses triggered upon sensing of WT HIV-infected cells. Noticeably, BST2 blocking experiments using anti-BST2 Abs (Fig 5) or soluble ILT7 (Fig 9) phenocopied this result. Second, a potential difference in the efficiency of virus transmission from infected T cell to pDCs cannot explain why depletion of ILT7 in pDCs eliminates the modulatory effect of Vpu on IFN-I production nor why a differential activation of ILT7 by WT or dU HIV-expressing cells can be observed with a reporter cell line that is not susceptible to HIV infection (Fig 9).

Our results are more consistent with a mechanism of innate immune evasion whereby Vpu regulates the levels of BST2 molecules on HIV-producing cells that are capable of engaging the ILT7 inhibitory receptor on pDCs (Fig 10). Our immuno-localization studies in HIV-infected T cells indeed reveal the presence of a residual pool of surface BST2 that is found outside viral assembly sites. In the absence of Vpu, such a pool could not be detected since a very large proportion of surface BST2 molecules were found to co-localize with assembling viruses (Fig 6). While the exact nature of this residual surface BST2 pool remains to be precisely defined, one possibility is that it could be generated by a displacement of BST2 molecules from sites of viral assembly by Vpu. Indeed, it was recently shown that in addition to causing BST2 sequestration to internal compartments and downregulation from the cell surface, Vpu had the ability to directly bind and displace BST2 from nascent virions at the plasma membrane [30,31]. This property would enable Vpu to exhibit residual BST2 antagonist activity in the absence of BST2 downregulation.

Two isoforms of human BST2 can be generated by alternative translation initiation [33]. These isoforms display distinct biological activities and have the ability to form homodimers and heterodimers. The short isoform, which lacks a dual-tyrosine-based sorting motif, phospho-tyrosine sites and potential ubiquitination acceptor residues present in the long isoform, is unable to induce NFκB activation and is significantly more resistant to Vpu-mediated downregulation and degradation [33,34]. Since both of these isoforms retain a functional ectodomain capable of interacting with ILT7, we examined whether they could have evolved to ensure both a potent restriction of invading viruses as well as an effective regulation of viral innate immune sensing and its resulting antiviral and proinflammatory responses. Thus, by preferentially targeting the long BST2 isoform for downregulation and degradation, HIV would not only overcome BST2 antiviral and signaling activities, but also maintain the negative signaling on IFN-I production exerted by the short BST2 isoform via ILT7. Our immuno-localization analysis reveals that both isoforms strongly co-localize with assembling virions in the absence of Vpu, and as such would be occluded from potential interactions with ILT7 on pDCs. A surprising finding from these studies was the effective capacity of Vpu to displace the short BST2 isoform from viral assembly sites and to overcome its tethering activity despite its resistance to Vpu-mediated downregulation (S3B, S3C and S3D Fig). This finding suggests that the sensitivity of short BST2 to Vpu antagonistic activity in infected T cells may be more significant than anticipated from previous data generated in transiently transfected HEK 293T cells [33]. In fact, both isoforms could be detected outside viral assembly sites in the presence of Vpu, suggesting that in both contexts a pool of surface BST2 molecules would potentially be accessible for interaction with ILT7. However, given their different surface levels and frequency of localization outside viral assembly sites, it is likely that the short BST2 isoform will be over-represented at the surface of HIV-1 WT-infected cells. Unexpectedly, despite these differences, we could not detect any differential repression of IFN-I production when pDCs were co-cultured with infected SupT1-shortBST2 or SupT1-longBST2 cells. In both cases, the presence of Vpu in virus-producing cells attenuated production of IFN-I by pDCs to a similar extent (S5 Fig). While this apparent discrepancy may result from the already potent repression observed with BST2-expressing SupT1 cells (Fig 7D and 7E), it may also well be possible that our co-culture assay is not sensitive enough to detect such a difference in repression or that expression levels of single isoforms in the cell lines may be a contributing factor. Nevertheless, our data indicate that in WT HIV-infected T cells, significant amounts of short BST2 homodimers as well as a smaller fraction of long BST2 homodimers (and possibly heterodimers) are re-distributed outside virus assembly sites in the presence of Vpu where they would be potentially accessible to engage the ILT7 pDC receptor. Whether BST2 molecules remaining outside viral assembly sites interact with ILT7 at contact zones of virological synapses between infected donor T cells and target pDCs is an interesting question that requires further study.

Our results suggest that through a sophisticated targeted regulation of specific BST2 isoforms involving surface downregulation and/or exclusion from viral assembly sites, Vpu could promote HIV-1 release while at the same time interfering with pDC antiviral responses through ILT7 activation (Fig 10). Interestingly, a recent study analyzed the sensitivity of the long and short BST2 isoforms to counteraction by primate lentiviruses and found that the differential targeting of BST2 isoforms by Vpu appears a specific property of proteins encoded by pandemic group M HIV-1 [34]. In contrast, both isoforms of the human and rhesus macaque BST2 demonstrated similar sensitivities to viral countermeasures by HIV-2 and SIVmac, respectively. It is interesting to note that the viral antagonists used by HIV-2 (Env) and SIVmac (Nef) counteract BST2 by a non degradative process, which involves removal of the protein from the cell surface through enhanced internalization and intracellular sequestration [39–42]. Vpu proteins encoded by SIV strains from African guenons as well as a Vpu protein from a recently isolated highly pathogenic HIV-1 group N from Togo (N1.FR.2001) [43] antagonized both isoforms, although in the latter case, N1.FR.2001 Vpu was unable to induce the degradation nor the downregulation of either isoforms [34]. Moreover, it was recently shown that HIV-1 group O strains have evolved to use Nef and not Vpu to counteract human BST2 [44]. However, consistent with their ability to target a domain in the N-terminal cytosolic region of human BST2 that is missing in short BST2, group O Nef did not antagonize the short isoform. Thus, primate lentiviruses diverge in their ability to differentially target BST2 isoforms with Vpu proteins from the pandemic group M showing a unique and conserved ability to counteract differently the short and long BST2 isoforms. Whether this targeted regulation of BST2 isoforms by Vpu may have contributed to the successful spread of HIV-1 group M at least in part by allowing a suppression of innate sensing by pDCs, remains an interesting possibility that will require further studies.

Activation of the ILT7–FcεRIγ complex by BST2 was found to initiate the ITAM-mediated activation of Src and Syk kinases in pDCs, a condition that ultimately inhibits production of IFN-I and other proinflammatory cytokines [25]. Our binding and functional studies support previous results from Cao and colleagues showing that BST2 binds and potently activates ILT7. Recently, the role of the ILT7-BST2 pair in the modulation of IFN-I production by pDCs has been challenged [45]. This study showed that treatment of PBMCs with the BST2 mAb 26F8 failed to enhance IFN-I release following TLR9 agonist treatment, despite the ability of 26F8 to block ILT7/BST2 binding. While these findings appear to challenge the physiological relevance of the BST2/ILT7 interaction, there are several possible alternative explanations for these results. The most important among these relates to the experimental system used in that study. In contrast to our co-culture system where an initial contact between infected T cells and pDCs is required for sensing to occur, treatment of PBMCs with TLR9 agonists activates pDCs directly without the need for cell-to-cell contact. Interestingly, all our attempts to differentially impact pDC ability to produce IFN-I through co-coculture with CD4+ T cells expressing or not BST2 in presence of TLR agonists were unsuccessful. In contrast, a significant reduction of IFN-I production by pDCs treated with TLR7 agonist could be observed upon co-cultures with HEK293T cells overexpressing BST2 (Fig 7B), suggesting that the concentration of BST2 at the cell-to-cell contact might also be an important factor driving the activation of ILT7. Furthermore, since the interaction between ILT7 and BST2 needed to be stabilized with cross-linking agents *in cellulo* (Fig 8C, 8D and 8E) and that close cell-to-cell contact was required to achieve activation of ILT7 by BST2-expressing transformed or cancer cells [25], it remains to be demonstrated whether contacts between activated pDCs and bystander normal T cells (as opposed to transformed or infected cells) would be frequent and sustained enough to engage the BST2/ILT7 negative feedback system. Indeed, it was reported that pDCs preferentially form conjugates with herpes simplex virus (HSV)-infected cells compared to uninfected cells [46]. Clearly, more studies will be required to define the determinants that drive BST2/ILT7 interactions at infected T cell/pDC contacts.

Studies in humanized (hu) mice indicated that Vpu-mediated BST2 antagonism promoted HIV-1 replication and propagation *in vivo*, especially at early times post-infection when the predominant mode of viral transmission is likely to be by cell-free viruses [47,48]. Under these conditions, Vpu appeared to be important in ensuring the efficient initial viral expansion that is most likely necessary to enable dissemination to local lymphoid tissues and establishment of infection [49]. Given the requirement of Vpu-mediated BST2 antagonism for efficient innate immune evasion, it is tempting to speculate that control of pDC antiviral responses during early infection could also be important to ensure sufficient local viral expansion so that widespread dissemination could occur. Indeed, consistent with their role as a major source of IFN-I, pDCs were found to effectively suppress HIV replication in hu-mice. Depletion of pDCs prior to HIV infection in that model prevented induction of IFN-I and IFN-stimulated genes (ISGs) and increased viral replication and dissemination [5]. Therefore, by limiting IFN-I and proinflammatory cytokines production by pDCs in the initial phase of infection, Vpu may contribute to the increased transmission fitness of T/F viruses by enabling efficient viral spread in an environment where expression of BST2 and other ISGs remain low [49].

Clearly, a better understanding of the mechanisms underlying Vpu-mediated inhibition of IFN-I production by pDCs during sensing of HIV infected cells may provide important insights into viral transmission and pathogenesis during acute infection

## Materials and Methods

### Antibodies and reagents

Rabbit polyclonal anti-BST2, anti-Vpu, and anti-p17 antibodies (Abs) were previously described [32,50,51]. Mouse anti-BST2 (mAb 26F8), anti-ILT7 (17G10.2) and their respective isotype controls were purchased from eBiosciences. The following mouse mAb: anti-HA (HA.11 Clone 16B12, formerly Covance), anti-CD3_Pacific Blue, anti-ILT7_PE, anti-ILT7_alexa647 and anti-CD4_PerCP/Cy5.5 were purchased from Biolegend, while anti-CD14_PE/Texas Red and anti-CD303 (BDCA-2)_APC were purchased from Caltag and Miltenyi, respectively. Anti-HIV-1 gp120 Monoclonal 2G12 (anti-Env) was obtained through the NIH AIDS Reagent Program [52–56]. All secondary Abs used for flow cytometry and western blot were purchased from Life Technologies and BioRad, respectively. Goat anti-human IgG was obtained from Abcam. Human rIFN-α2a was purchased from PBL. TLR7 and 9 agonists, TLR9 antagonist and their respective controls were obtained from InvivoGen: TLR7 agonists Imiquimod (final concentration: 2.5 μg/ml) and R848 (10 μg/ml); TLR9 agonist ODN 2216 CpG-A (5 μM); TLR9 antagonist or its control (ODN_TTAGGG_ and ODN_Ctrl_, 100nM). TLR7/8/9 antagonist or its control, were kindly provided by Idera Pharmaceuticals (final concentration: 500 nM) [27]. Fusion (T-20, 0.5 μM) and reverse transcription (3TC, 300 μM) inhibitors were obtained from the NIH AIDS Reagent Program, Division of AIDS, NIAID. Various types of siRNAs and transfection conditions were tested in order to select a combination that did not trigger IFN-I production by pDCs. Among the tested conditions, ON-TARGETplus siRNAs transfections using Oligofectamine (Invitrogen) was selected as it effectively depleted the target gene, did not trigger spontaneous IFN-I production after transfection, and displayed low toxicity in treated pDCs. ON-TARGETplus SMARTpool siRNAs targeting ILT7 and negative control C8b were obtained from Thermo Scientific and were transfected with Oligofectamine reagent according to the manufacturer’s recommendations. Purity and viability of transfected pDCs after co-culture was examined by flow cytometry (Forward Scatter / Side Scatter and BDCA-2+) and assessment of IFN-I production capacity.

### Cell lines and plasmids

SupT1 and MT4 T cells were obtained from the NIH AIDS reagents program [57] while HEK293T and HEK-blue human IFN reporter cell lines were obtained from ATCC and InvivoGen, respectively. HEK293T cells were transiently transfected using lipofectamine 2000 (Invitrogen Inc). The ILT7 NFAT-GFP reporter cell lines were a generous gift from Dr. Yong-Jun Liu [24]. In the ILT7+ NFAT-GFP reporter mouse cell line (CT550), which expresses ILT7 and Fc*ϵ*RIγ, GFP is driven by an NF-AT promoter (NFAT-GFP) and results in GFP expression in response to ILT7 surface ligation. The negative control ILT7- NFAT-GFP reporter mouse cell line (CT59Fc) expresses Fc*ϵ*RIγ and NFAT-GFP, but not ILT7.

A panel of full-length transmitted/founder (T/F) HIV-1 infectious molecular clones (Cat #11919) was obtained through the NIH AIDS Reagent Program [58–61]. Except for T/F CH077, all viruses used were derived from a pNL4.3 construct (X4 WT), in which the *nef* gene is followed by an internal ribosome entry site that allows expression of GFP (pNL4.3-GFP) [62]. The Vpu-deficient mutant (dU) was generated by subcloning a SalI-KpnI fragment from the Vpu-defective pNLVpuDEL1 [63] into pNL4.3-GFP. The virus encoding a TM domain mutant of Vpu (Vpu A_10-14-18_L) attenuated in its ability to bind and antagonize BST2 [28] was generated by site-directed mutagenesis from pNL4.3-GFP. The infectious CCR5-tropic GFP-marked proviral DNA (pNL4.3-Ada-GFP; R5 WT) and Vpu-deficient mutant (R5 dU) were previously described [48]. Briefly, the pNL4.3 backbone was rendered CCR5-tropic by transferring the SalI-BamHI fragment from a well-characterized CCR5-tropic construct Ada (NLHXADA), which encodes Vpu and Env proteins from ADA [64]. GFP-marked NL4.3 viruses expressing T/F Suma Vpu (pNL-Suma) or T/F CH077 Vpu (pNL-77) were generated using the overlapping PCR method. PCR fragments amplified from pNL4.3 provirus, from the Sal I site to the beginning of Vpu (ATG) and from the end of Vpu (stop codon) to the BamH1 site in *env*, were fused to PCR-generated DNA fragments encompassing the Vpu open reading frame from either T/F Suma or CH077 proviruses. The resulting chimeric fragments were digested with Sal I and BamH1 and cloned back into pNL4.3 backbone. The sequence of the Vpu-Env region of all provirus constructs used was validated by automated sequencing.

ILT7 expressing plasmid, pMX-Puro-HA-ILT7, and BST2-Fc expressing plasmid were a generous gift from Dr. Wei Cao (Department of Immunology, University of Texas, MD Anderson Cancer Center, Houston) [25]. The BST2-Fc construct expresses a soluble form of BST2 lacking the cytoplasmic tail, the transmembrane region and the GPI anchor. The HA-tagged ILT7 open reading frame was sub-cloned from its original backbone to pcDNA3.1. soILT7-HA was amplified by PCR from the pcDNA3.1-HA-ILT7 plasmid. The encoded N-terminal signal peptide and HA-tag were maintained but the amino acid (aa) sequence was truncated after residue 435 by adding a premature stop codon right before the TM domain (HA-ILT7). The soILT7-HA DNA fragment was inserted back into the pcDNA3.1 backbone. For Surface Plasmon Resonance (SPR) analysis, the synthetic codon-optimized ILT7 gene (Eurofins Genomics) encoding residues 24 to 435 (bacILT7) was cloned into the transfer vector pFL, followed by Tn7-based transposition into the EMBacY bacmid to generate a recombinant baculovirus [65]. The extracellular domain of BST2 (residues 47 to 159) was cloned into the pBADM30 expression vector in order to construct a His-tagged GST-N-terminus fusion protein. The original plasmid for subcloning of BST2-dGPI, Tetherin delGPI, was a generous gift from Dr. Paul Bieniasz [17]. Short (delta Met1) or long (delta Met13) BST2 were generated by PCR and fused to a weak kozak sequence. WT BST2, BST2-dGPI, short BST2 and long BST2 open reading frames were cloned into the pcDNA3.1 backbone for transient transfections and in pLenti-CMV/TO_Puro_DEST (Addgene) for the generation of stable cell lines.

MT4 cells were transduced using lentiviral vector particles containing shRNA targeting BST2 (Clone ID: TRCN0000107018, from OpenBiosystem) or control shRNA (target sequence: 5’CAACAAGATGAAGAGCACCAA3’). SupT1 cells stably expressing the different BST2 isoforms (WT BST2, dGPI, short and long BST2) as well as control SupT1 cells (empty) were established by lentiviral vector transduction, based on pLenti-CMV/TO_Puro_DEST plasmids. Briefly, recombinant lentiviral particles were generated by transfecting HEK293T cells with the lentiviral vector together with psPAX2, a plasmid encoding HIV-1 Gag/Pol, Tat and Rev, as well as with pVSVg, a vector expressing the G glycoprotein of vesicular stomatitis virus (VSV), as previously described [66]. Two days post-transfection, the culture media containing the lentiviral particles was used to transduce SupT1 or MT4 cells. Cell lines expressing short or long BST2 isoforms were evaluated by Western Blot following immunoprecipitation and PNGase F glycosidase treatment to remove carbohydrate modifications (New England Biolabs, Inc.). Briefly, cell lysates were immunoprecipitated with rabbit anti-BST2 Abs, digested with PNGase F overnight at 37°C and analyzed by 16% Tris-Tricine SDS-PAGE.

### HIV-1 virus production and infection

Infectious HIV-1 viruses T/F CH077, GFP-marked NL4.3 or VSV-G-pseudotyped NL4.3-Ada virus derivatives were generated by lipofectamine transfection of HEK293T cells. Supernatants containing virus were harvested 2 days post transfection, clarified, pelleted by ultracentrifugation and titrated using the TZM-bl indicator cells as described previously [51].

### Virus particle release assay

Release of virus particle was assessed by Western blot as described previously [67]. Viral particle release efficiency was evaluated by determining the ratio of virion-associated Gag (p24) signal over the total intracellular Gag (p24 + p55) signal measured by scanning densitometry analysis of Western blots. Viral release efficiency was normalized to the value obtained in cells infected with WT virus, which was set at 100%.

### Preparation of primary CD4+ T cells and PBMCs/pDCs

Peripheral blood samples were obtained from healthy adult donors who gave written informed consent in accordance with the Declaration of Helsinki under research protocols approved by the research ethics review board of the IRCM. PBMCs were isolated by Ficoll-Paque centrifugation (GE Healthcare) and cultured in RPMI-1640 media supplemented with 10% FBS. CD4+ T lymphocytes were isolated by negative selection using a CD4+ T Cell Isolation Kit (Miltenyi Biotec). Enriched CD4+ T cells were then activated using PHA-L (5 μg/mL) for 48 h and maintained in RPMI-1640 complete medium supplemented with IL-2 (100 U/mL). Activated primary T cells were infected 5 days post-isolation. Human pDCs were enriched by negative selection using the Diamond Plasmacytoid Dendritic Cell Isolation Kit II (Miltenyi Biotec). Surface phenotyping was carried-out by flow cytometry as described.

### Co-culture assay with PBMCs/pDCs

Two days prior to co-culture, T cells were infected with T/F CH077 or different GFP-marked pNL4.3 viruses, at different MOIs. Infection rates were calculated by measurement of GFP+ or intracellular p24+ cells by flow cytometry, as previously described [48]. Cultures with a range of 20–50% infected cells were subsequently used for co-cultures. Target and donor cell were mixed at a ratio of 3:1 (PBMC:T cell) or 1:5 (pDC:T cell) in a final volume of 250 μl and cultured in U-bottom 96-well plates for 18–22 h. Fusion (T-20, 0.5μM) or reverse transcription (3TC, 300μM) inhibitors were added to infected MT4 or to freshly isolated PBMCs, respectively, for 1 h prior to co-culture. For TLR antagonist experiments, freshly isolated PBMCs were pre-treated with either TLR9 antagonist or its control (ODN_TTAGGG_ and ODN_Ctrl_, 100nM) or TLR7/8/9 antagonist or its control (500nM) for 1 h prior to co-culture with infected cells. Agonist treatments (ODN_2216,_ 50fM for TLR 9 and R848 10ug/ml for TLR7) were used as positive controls. For BST2 blocking experiments, mock or infected MT4 cells were pre-treated for 30 min with anti-BST2 rabbit polyclonal or pre-immune Abs at 37°C, prior to the 18h co-culture with PBMCs. Alternatively, HEK293T cells were transfected with plasmids (pLenti-CMV/TO_Puro_DEST) encoding for WT BST2 or BST2-dGPI 2 days prior to co-culture with PBMCs. After 6 h of co-culture, TLR7 agonist Imiquimod was added to a final concentration of 2.5 μg/ml and cells were kept in co-culture for an additional 18 h. In all conditions, co-cultures were then transferred to a V-bottom 96-well plate, and centrifuged for 5 min at 400g. Supernatants were then used to quantify the amounts of IFN-I produced as described in the supplemental information. Each experimental replicate (n) was performed using cells from a different donor.

### Surface antigen staining and flow cytometry analysis

BST2 cell-surface staining and flow cytometry analysis of live cells was performed as previously described [51]. In all histograms shown, mean fluorescence intensity (MFI) values are shown for each sample. Surface phenotyping was carried-out using multi-parametric surface flow cytometry staining. Briefly, freshly isolated PBMCs or pDCs (2 x 10^6^ cells/ml and 5 x 10^4^ cells/ml, respectively) were washed with cold PBS/EDTA/FBS, blocked with human IgG, and stained with the appropriate fluorochrome-conjugated surface cellular marker Abs for 60 min at 4°C. The CD4+ subpopulations of T cells were defined as CD3+/CD4+/CD14-. The monocytes were defined as CD3-/CD14+, while the pDC population was defined as CD3-/CD14-/BDCA-2+/ILT7+. Cells were washed, re-suspended in PBS and analyzed using a Cyan flow cytometer with FlowJo software (Treestar).

### Quantification of IFN-I

Detection of bioactive human IFN-I was performed using reporter cell line HEK-Blue IFN-α/β (InvivoGen) as previously described [66]. IFN-I concentration (U/ml) was extrapolated from the linear range of a standard curve generated using known amounts of IFN-I.

### Confocal microscopy

Primary CD4+ T cells, MT4 and SupT1 cell lines were infected with VSV-G-pseudotyped NL4.3-Ada (WT or dU) viruses. Forty-eight hours post-infection, cells were immunostained with anti-BST2 and anti-Env Abs for 45 min at 4°C prior to extensive washes. Cells were then plated on polyD-lysine-treated coverslips and fixed for 30 min in 4% PFA. Viral matrix p17, a product of Gag polyprotein cleavage by viral protease during viral budding at the surface of the infected cells, was used as marker of assembling HIV-1 particles and was detected with a specific antibody that does not recognized immature Gag products [32]. To detect p17 fixed cells were permeabilized in Triton 0.2% for 5 min, incubated for 2 h at 37°C in 5% milk-PBS containing anti-p17 Abs, washed and incubated with the appropriate secondary Ab for 30 min at room temperature. All analyses were acquired using a 63× Plan Apochromat oil immersion objective with an aperture of 1.4 on an LSM710 Observer Z1 laser scanning confocal microscope coupled with a Kr/Ar laser (Zeiss). Surface BST2 was quantified by measuring raw integrated signal density using ImageJ software on manually selected cells.

### BacILT7 production and surface plasma resonance analysis

The supernatant of SF21 insect cells secreting bacILT7 was collected 4 days post infection, dialyzed against buffer A (20 mM Tris, 150 mM NaCl, pH 7.2) and concentrated 2-fold. The supernatant was then applied to a nickel-affinity chromatography (Qiagen) column. The column was washed sequentially with buffer A containing 10, 50 and 70 mM imidazole, followed by elution of bacILT7 with buffer A containing 300 mM imidazole. The eluted fractions were pooled and dialyzed extensively against buffer B (20 mM Tris, 150 mM NaCl, 10% glycerol). Analytical size exclusion chromatography showed that the majority of the protein eluted in a peak at 13 mL from a Superdex 200 column. BacILT7 was dialyzed against HBS-PE and cleared by centrifugation at 100,000 g for 20 min. GST-BST2 was expressed in *E*. *coli* Rosetta (DE3) cells (Novagen) and purified in buffer C (20 mM Tris, 100 mM NaCl, pH 7.5) by nickel-affinity chromatography (Qiagen) followed by size-exclusion chromatography on a Superdex 200 column (GE Healthcare) in buffer D (20 mM HEPES, 100 mM NaCl, 10 mM EDTA, pH 7.5). Surface plasma resonance was performed on a Biacore 3000 (GE Healthcare) system using HBS-PE as a running buffer (10 mM HEPES pH 7.5, 150 mM NaCl, 3 mM EDTA and 0.005% Tween-20). GST-BST2 was diluted to 5 μg/ml in 10 mM sodium acetate (pH 4) buffer and covalently immobilized to the surface of a CM5 sensor chip by amine coupling according to the manufacturer’s instructions, yielding an R_ligand_ of 6550 RU. A reference flow cell was generated by amine coupling of GST alone (R_ligand_ = 1028). BacILT7 was serially diluted into running buffer and passed over the chip at a flow rate of 10 μl/min. The response from the GST-coated reference cell was subtracted from the response resulting from specific binding to the target protein. Regeneration of the sensor chip was achieved with 10 mM HCl for 60 seconds. The spikes present in the sensorgrams are due to a delay in the bulk refractive index change between the flow cells, which is exacerbated by the 10 μl/min flow rate. Data were analyzed with the BIAevalution software version 4.1.

### Production of BST2-Fc and soILT7-HA

HEK293T cells were transiently transfected with a control empty plasmid, with BST2-Fc or with soILT7-HA expressing-plasmid using lipofectamine 2000 (Invitrogen Inc). Cell culture medium was replaced 6 h post transfection with serum-free medium (DMEM supplemented with Nutridoma-SP (Roche, Life Science)). Supernatant containing the soluble protein as well as control were collected 48 h post transfection, clarified by centrifugation (400g, 5 min) and filtered through a 0.2 μm pore size membrane.

### Analysis of BST2-ILT7 interaction using flow cytometry

HEK293T cells were transfected with an empty plasmid or with a construct encoding the full length ILT7. Cells were detached and incubated with control supernatant or with BST2-Fc-containing supernatant (approximately 300 μg/ml) for 30 min at 4°C, prior to crosslinking with DTSSP (3,3´-dithiobis [sulfosuccinimidylpropionate], Thermo Scientific) for 30 min at room temperature. The crosslinking reaction was stopped by addition of 1 M Tris, pH 7.5. Cells were washed and then stained for surface BST2-Fc using on one hand rabbit polyclonal anti-BST2 serum and goat anti-rabbit IgG coupled with Alexa 633 to label the BST2 portion and on the other hand anti-human IgG coupled with Alexa 633 to label the Fc fragment of the recombinant protein. Cells were analyzed using a Cyan ADP flow cytometer and FlowJo software (Treestar).

### *In situ* proximity ligation assay

*In situ* proximity ligation assay (PLA) was performed using the Duolink kit 613 (Sigma Aldrich). Briefly, ILT7+ NFAT-GFP or ILT7- NFAT-GFP reporter cells were incubated with control supernatant (CTRL sup) or with BST2-Fc-containing supernatant (BST2-Fc sup, approximately 300 μg/ml) prior to crosslinking with DTSSP for 30 min at room temperature. The crosslinking reaction was stopped by addition of 1M Tris, pH 7.5. Cells were washed, adhered on poly-L Lysine slides, fixed using a 4% PFA solution and blocked using the blocking solution provided in the PLA kit. Fixed cells were then incubated with the following Abs: mouse mAb antibody against ILT7 or rabbit polyclonal serum against BST2. The Duolink system provides oligonucleotide-labeled secondary Abs (PLA probes) to each of the primary Abs that, in combination with a DNA amplification-based reporter system, generate a signal only when the two primary Abs are in close proximity (less than 40 nm). Following the manufacturer’s recommendation, after addition of the PLA probes, the oligonucleotides were ligated and amplified using the ligase and polymerase provided. Finally, nuclei were counter-stained using DAPI. The signal from each detected pair of primary Abs was visualized as a red spot using fluorescence confocal microscopy.

### Activation of ILT7 using ILT7+ NFAT-GFP reporter cells

Standard ELISA plates were sterilized by UV treatment. Plates were treated with either anti-ILT7 Abs or BST2-Fc. For treatment with ILT7 Abs, Alexa647-conjugated Anti-ILT7 Abs (diluted in PBS to 1 μg/ml) were allowed to adhere to plates for 18 h at 4°C. For BST2-Fc treatment, plates were first coated with goat anti-human IgG (diluted in PBS to 10 μg/ml) for 2 h at 4°C, washed with PBS and incubated with BST2-Fc-containing supernatant (approximately 300 μg/ml) for an additional 16 h at 4°C. The following day, ILT7+ NFAT-GFP reporter cells were added and plates were incubated for 18 h at 37°C. To test the effect of unbound ILT7 Abs (soluble ILT7 Abs), Alexa647-conjugated Anti-ILT7 Abs (diluted in media to 1 μg/ml) were added directly to ILT7+ NFAT-GFP reporter cells prior to an overnight incubation. Cells were collected and analyzed by flow cytometry for GFP expression. For ILT7 activation experiments involving BST2-expressing cells, BST2-expressing or control cell lines (50,000 cells/well) were plated in 24 well plates 18–24 h prior to co-culture with either ILT7+ NFAT-GFP or control ILT7-NFAT-GFP reporter cells (100,000 cells/well). The co-cultures were maintained for an additional 18–24 h, at which time samples were analyzed by flow cytometry for GFP expression. For blocking experiments, anti-ILT7 Abs (10 μg/ml) were added to reporter cells or anti-BST2 (30 μl of polyclonal rabbit serum) to BST2-expressing or control cells for 1 h prior to co-culture.

### Statistical analysis

Statistical analysis was performed using repeated measures ANOVA, with Bonferroni’s multiple comparison test or two-tailed paired Student’s *t*-tests. A p value of <0.05 was considered significant. The following symbols were used throughout the manuscript: *** p<0.001, ** p<0.01, * p<0.05, ns not significant (p>0.05).

## Supporting Information

S1 Fig Vpu promotes HIV-1 viral release and BST2 surface down-modulation in infected MT4 cells. **(A-D)** MT4 cells were mock-infected (m) or infected with GFP-marked NL4.3 (WT or dU) as indicated. **(A)** Cells and virion-containing supernatants were analyzed for the presence of Gag proteins and Vpu by Western blot, 48 hours post infection (hpi), as indicated. **(B)** Relative virus particle release efficiency was calculated as described in Materials and Methods and normalized to the value obtained with the WT virus, which was set at 100% (n = 3). **(C)** Flow cytometry analysis of surface BST2 in GFP-positive MT4 cells infected with WT (dashed grey histogram) or dU (solid black histogram), 48 hpi. Mean fluorescence intensity (MFI) values are indicated for each sample (staining using pre-immune rabbit serum, PI, shaded grey histograms). **(D)** Relative BST2 surface expression after infection with the indicated HIV viruses (n = 4). Percentage MFI were calculated relative to dU HIV-producing cells (100%). **(E-G)** MT4 cells were infected with GFP-marked NL4.3 virus lacking Vpu (dU) or encoding either NL4.3 Vpu (WT), T/F Suma Vpu (pNL-Suma) or T/F CH077 Vpu (pNL-77). **(E)** Cells and virion-containing supernatants were analyzed by Western blot as described in panel A. Note that detection of T/F CHO77 Vpu required a longer exposure since rabbit polyclonal anti-BST2 Abs were inefficient at recognizing this Vpu variant. **(F)** Relative virus particle release efficiency was determined as described in panel B (n = 2). **(G)** Surface BST2 expression was evaluated by flow cytometry 48 hpi as described in panel C. Error bars represent standard deviations (SD).
(PDF)

S2 Fig Virus release assays in BST2-depleted MT4 cell lines and phenotypic analysis of the VpuA10-14-18L TM mutant virus (A-B) Control (MT4-shNT) or BST2-depleted (MT4-shBST2) MT4 cells were mock-infected, or infected with GFP-marked NL4.3 WT or dU viruses. **(A)** Cells and virion-containing supernatants were analyzed by Western blot as described in S1 Fig. The absolute amounts of virus released in each condition was estimated by densitometry scanning of the virion-associated p24 signal and is indicated under the blot as arbitrary densitometric unit (adu). **(B)** Relative virus particle release efficiency was determined as described in S1 Fig (n = 3). **(C-F)** MT4 cells were mock-infected or infected with GFP-marked NL4.3 WT, dU or VpuA10-14-18L TM mutant viruses. **(C)** Cells and virion-containing supernatants were analyzed by western blot as described in S1 Fig. **(D)** Relative virus particle release efficiency was determined as described in S1 Fig (n = 3). **(E-F)** The indicated MT4 donor cells were co-cultured with PBMCs. After 24 h, levels of IFN-I released in supernatants were measured. A representative example of absolute levels **(E)** or relative percentages **(F)** of IFN-I production after co-culture of infected MT4 cells with PBMCs are shown. The amount of IFN-I released by PBMCs in contact with dU HIV-infected cells was set at 100% (n = 12). Repeated measures ANOVA with Bonferroni’s multiple comparison tests was used (*** p<0.001, ns not significant (p>0.05)). Error bars represent standard deviations (SD).
(PDF)

S3 Fig Infection of primary CD4+ T cells and SupT1 cell lines expressing the short or long BST2 isoforms. **(A)** BST2 from SupT1 cells expressing either long or short isoforms was immunoprecipitated, treated with PNGase and analyzed by Western blot. As controls, BST2 from IFN-treated and untreated SupT1 and MT4 cells were similarly analyzed. * represent the Ab heavy chain and was used as loading control. **(B-D)** SupT1-shortBST2 and SupT1-longBST2 cells were mock-infected (m) or infected with NL4.3-GFP WT or dU viruses. **(B)** Surface BST2 expression was evaluated by flow cytometry 48 hpi, as described in S1 Fig. **(C)** Cells and virion-containing supernatants were analyzed by western blot as described in S1 Fig. The absolute amount of virus released in each condition was estimated by densitometry scanning of the virion-associated p24 signal and is indicated under the blot as arbitrary densitometric unit (adu). **(D)** Relative virus particle release efficiency was determined as described in S1 Fig (n = 3). HIV-1 WT release efficiency in SupT1-longBST2 was set at 100%. Error bars represent standard deviations (SD). **(E-F)** Primary CD4+ T cells and SupT1-shortBST2 cells were mock-infected (mock) or infected with VSV-G-pseudotyped NL4.3-Ada-GFP WT or dU viruses. **(E)** Infected primary CD4+ T cells were stained with anti-BST2 Abs (blue), fixed, permeabilized and then sequentially stained with anti-p17 Abs (red). A representative example of multiple cells is shown. **(F)** Infected primary CD4+ T cells and SupT1-shortBST2 cells were stained with anti-BST2 Abs (blue) and 2G12 anti-Env Abs (red). A representative example is shown. White bar = 10 μm.
(PDF)

S4 Fig Effect of Vpu during infection of SupT1 cells expressing BST2 or a BST2 GPI anchor mutant. SupT1-Empty, SupT1-BST2 and SupT1-BST2-dGPI cells were mock-infected or infected with GFP-marked NL4.3 WT or dU viruses. **(A)** Surface BST2 expression was evaluated by flow cytometry 48 hpi as described in S1 Fig. **(B)** Cells and virion-containing supernatants were analyzed by western blot as described in S1 Fig. The absolute amounts of virus released in each condition was estimated by densitometry scanning of the virion-associated p24 signal and is indicated under the blot as arbitrary densitometric unit (adu). **(C)** Relative virus particle release efficiency was determined as described in S1 Fig (n = 3). HIV-1 WT release efficiency in SupT1-BST2 was set at 100%. Error bars represent standard deviations (SD).
(PDF)

S5 Fig Comparable levels of IFN-I are produced upon sensing of HIV-infected cells expressing short or long BST2. SupT1 (no BST2), SupT1-longBST2 (long BST2) and SupT1-shortBST2 (short BST2) cells were mock-infected or infected with GFP-marked NL4.3 WT or T/F CH077 viruses for 48 h prior to co-culture with PBMCs. After 24 h of co-culture, levels of IFN-I released in supernatants were measured. A representative example of absolute levels (A) or relative percentages (B) of IFN-I detected after co-culture of WT or dU HIV-1-infected SupT1 donor cells with PBMCs. The amount of IFN-I released by PBMCs in contact with NL4.3- or CH077-infected SupT1-BST2 cells in the absence of BST2 was set at 100%. Repeated measures ANOVA with Bonferroni’s multiple comparison test was used (n >3). Error bars represent SD. ns- not significant.
(PDF)

## References

[1] Swiecki M, Colonna M (2010) Unraveling the functions of plasmacytoid dendritic cells during viral infections, autoimmunity, and tolerance. Immunol Rev 234: 142–162. 10.1111/j.0105-2896.2009.00881.x 20193017 PMC3507434

[2] Fitzgerald-Bocarsly P, Jacobs ES (2010) Plasmacytoid dendritic cells in HIV infection: striking a delicate balance. J Leukoc Biol 87: 609–620. 10.1189/jlb.0909635 20145197 PMC2858309

[3] Malleret B, Maneglier B, Karlsson I, Lebon P, Nascimbeni M, et al (2008) Primary infection with simian immunodeficiency virus: plasmacytoid dendritic cell homing to lymph nodes, type I interferon, and immune suppression. Blood 112: 4598–4608. 10.1182/blood-2008-06-162651 18787223

[4] Campillo-Gimenez L, Laforge M, Fay M, Brussel A, Cumont MC, et al (2010) Nonpathogenesis of simian immunodeficiency virus infection is associated with reduced inflammation and recruitment of plasmacytoid dendritic cells to lymph nodes, not to lack of an interferon type I response, during the acute phase. J Virol 84: 1838–1846. 10.1128/JVI.01496-09 19939930 PMC2812402

[5] Li G, Cheng M, Nunoya J, Cheng L, Guo H, et al (2014) Plasmacytoid dendritic cells suppress HIV-1 replication but contribute to HIV-1 induced immunopathogenesis in humanized mice. PLoS Pathog 10: e1004291 10.1371/journal.ppat.1004291 25077616 PMC4117636

[6] Lepelley A, Louis S, Sourisseau M, Law HK, Pothlichet J, et al (2011) Innate sensing of HIV-infected cells. PLoS Pathog 7: e1001284 10.1371/journal.ppat.1001284 21379343 PMC3040675

[7] Fonteneau JF, Larsson M, Beignon AS, McKenna K, Dasilva I, et al (2004) Human immunodeficiency virus type 1 activates plasmacytoid dendritic cells and concomitantly induces the bystander maturation of myeloid dendritic cells. J Virol 78: 5223–5232. 15113904 10.1128/JVI.78.10.5223-5232.2004 PMC400371

[8] Beignon AS, McKenna K, Skoberne M, Manches O, DaSilva I, et al (2005) Endocytosis of HIV-1 activates plasmacytoid dendritic cells via Toll-like receptor-viral RNA interactions. J Clin Invest 115: 3265–3275. 16224540 10.1172/JCI26032 PMC1253628

[9] Manches O, Munn D, Fallahi A, Lifson J, Chaperot L, et al (2008) HIV-activated human plasmacytoid DCs induce Tregs through an indoleamine 2,3-dioxygenase-dependent mechanism. J Clin Invest 118: 3431–3439. 10.1172/JCI34823 18776940 PMC2528911

[10] Schmidt B, Ashlock BM, Foster H, Fujimura SH, Levy JA (2005) HIV-infected cells are major inducers of plasmacytoid dendritic cell interferon production, maturation, and migration. Virology 343: 256–266. 16278001 10.1016/j.virol.2005.09.059

[11] Iwasaki A (2012) Innate immune recognition of HIV-1. Immunity 37: 389–398. 10.1016/j.immuni.2012.08.011 22999945 PMC3578946

[12] Martinelli E, Cicala C, Van Ryk D, Goode DJ, Macleod K, et al (2007) HIV-1 gp120 inhibits TLR9-mediated activation and IFN-{alpha} secretion in plasmacytoid dendritic cells. Proc Natl Acad Sci U S A 104: 3396–3401. 17360657 10.1073/pnas.0611353104 PMC1805537

[13] Chung NP, Matthews K, Klasse PJ, Sanders RW, Moore JP (2012) HIV-1 gp120 impairs the induction of B cell responses by TLR9-activated plasmacytoid dendritic cells. J Immunol 189: 5257–5265. 10.4049/jimmunol.1201905 23100517 PMC3504132

[14] Neil SJ (2013) The antiviral activities of tetherin. Curr Top Microbiol Immunol 371: 67–104. 10.1007/978-3-642-37765-5_3 23686232

[15] Hinz A, Miguet N, Natrajan G, Usami Y, Yamanaka H, et al (2010) Structural basis of HIV-1 tethering to membranes by the BST-2/tetherin ectodomain. Cell Host Microbe 7: 314–323. 10.1016/j.chom.2010.03.005 20399176 PMC2859121

[16] Kupzig S, Korolchuk V, Rollason R, Sugden A, Wilde A, et al (2003) Bst-2/HM1.24 is a raft-associated apical membrane protein with an unusual topology. Traffic 4: 694–709. 12956872 10.1034/j.1600-0854.2003.00129.x

[17] Neil SJ, Zang T, Bieniasz PD (2008) Tetherin inhibits retrovirus release and is antagonized by HIV-1 Vpu. Nature 451: 425–430. 10.1038/nature06553 18200009

[18] Van Damme N, Goff D, Katsura C, Jorgenson RL, Mitchell R, et al (2008) The interferon-induced protein BST-2 restricts HIV-1 release and is downregulated from the cell surface by the viral Vpu protein. Cell Host Microbe 3: 245–252. 10.1016/j.chom.2008.03.001 18342597 PMC2474773

[19] Casartelli N, Sourisseau M, Feldmann J, Guivel-Benhassine F, Mallet A, et al (2010) Tetherin restricts productive HIV-1 cell-to-cell transmission. PLoS Pathog 6: e1000955 10.1371/journal.ppat.1000955 20585562 PMC2887479

[20] Giese S, Marsh M (2014) Tetherin can restrict cell-free and cell-cell transmission of HIV from primary macrophages to T cells. PLoS Pathog 10: e1004189 10.1371/journal.ppat.1004189 24991932 PMC4081785

[21] Jolly C, Booth NJ, Neil SJ (2010) Cell-cell spread of human immunodeficiency virus type 1 overcomes tetherin/BST-2-mediated restriction in T cells. J Virol 84: 12185–12199. 10.1128/JVI.01447-10 20861257 PMC2976402

[22] Venkatesh S, Bieniasz PD (2013) Mechanism of HIV-1 virion entrapment by tetherin. PLoS Pathog 9: e1003483 10.1371/journal.ppat.1003483 23874200 PMC3715405

[23] Cao W, Bover L (2010) Signaling and ligand interaction of ILT7: receptor-mediated regulatory mechanisms for plasmacytoid dendritic cells. Immunol Rev 234: 163–176. 10.1111/j.0105-2896.2009.00867.x 20193018 PMC2919054

[24] Cao W, Rosen DB, Ito T, Bover L, Bao M, et al (2006) Plasmacytoid dendritic cell-specific receptor ILT7-Fc epsilonRI gamma inhibits Toll-like receptor-induced interferon production. J Exp Med 203: 1399–1405. 16735691 10.1084/jem.20052454 PMC2118323

[25] Cao W, Bover L, Cho M, Wen X, Hanabuchi S, et al (2009) Regulation of TLR7/9 responses in plasmacytoid dendritic cells by BST2 and ILT7 receptor interaction. J Exp Med 206: 1603–1614. 10.1084/jem.20090547 19564354 PMC2715090

[26] Miyagi E, Andrew AJ, Kao S, Strebel K (2009) Vpu enhances HIV-1 virus release in the absence of Bst-2 cell surface down-modulation and intracellular depletion. Proc Natl Acad Sci U S A 106: 2868–2873. 10.1073/pnas.0813223106 19196977 PMC2650357

[27] Kandimalla ER, Bhagat L, Wang D, Yu D, Sullivan T, et al (2013) Design, synthesis and biological evaluation of novel antagonist compounds of Toll-like receptors 7, 8 and 9. Nucleic Acids Res 41: 3947–3961. 10.1093/nar/gkt078 23396449 PMC3616729

[28] Vigan R, Neil SJ (2010) Determinants of tetherin antagonism in the transmembrane domain of the human immunodeficiency virus type 1 Vpu protein. J Virol 84: 12958–12970. 10.1128/JVI.01699-10 20926557 PMC3004320

[29] Miyagi E, Andrew A, Kao S, Yoshida T, Strebel K (2011) Antibody-mediated enhancement of HIV-1 and HIV-2 production from BST-2/tetherin-positive cells. J Virol 85: 11981–11994. 10.1128/JVI.05176-11 21917971 PMC3209280

[30] McNatt MW, Zang T, Bieniasz PD (2013) Vpu binds directly to tetherin and displaces it from nascent virions. PLoS Pathog 9: e1003299 10.1371/journal.ppat.1003299 23633949 PMC3635990

[31] Lewinski MK, Jafari M, Zhang H, Opella SJ, Guatelli J (2015) Membrane Anchoring by a C-terminal Tryptophan Enables HIV-1 Vpu to Displace Bone Marrow Stromal Antigen 2 (BST2) from Sites of Viral Assembly. J Biol Chem 290: 10919–10933. 10.1074/jbc.M114.630095 25759385 PMC4409254

[32] Dube M, Paquay C, Roy BB, Bego MG, Mercier J, et al (2011) HIV-1 Vpu antagonizes BST-2 by interfering mainly with the trafficking of newly synthesized BST-2 to the cell surface. Traffic 12: 1714–1729. 10.1111/j.1600-0854.2011.01277.x 21902775 PMC3955191

[33] Cocka LJ, Bates P (2012) Identification of alternatively translated Tetherin isoforms with differing antiviral and signaling activities. PLoS Pathog 8: e1002931 10.1371/journal.ppat.1002931 23028328 PMC3460627

[34] Weinelt J, Neil SJ (2014) Differential sensitivities of tetherin isoforms to counteraction by primate lentiviruses. J Virol 88: 5845–5858. 10.1128/JVI.03818-13 24623426 PMC4019096

[35] Jolly C, Sattentau QJ (2007) Human immunodeficiency virus type 1 assembly, budding, and cell-cell spread in T cells take place in tetraspanin-enriched plasma membrane domains. J Virol 81: 7873–7884. 17522207 10.1128/JVI.01845-06 PMC1951303

[36] Dube M, Bego MG, Paquay C, Cohen EA (2010) Modulation of HIV-1-host interaction: role of the Vpu accessory protein. Retrovirology 7: 114 10.1186/1742-4690-7-114 21176220 PMC3022690

[37] Galao RP, Pickering S, Curnock R, Neil SJ (2014) Retroviral retention activates a Syk-dependent HemITAM in human tetherin. Cell Host Microbe 16: 291–303. 10.1016/j.chom.2014.08.005 25211072 PMC4161388

[38] Galao RP, Le Tortorec A, Pickering S, Kueck T, Neil SJ (2012) Innate sensing of HIV-1 assembly by Tetherin induces NFkappaB-dependent proinflammatory responses. Cell Host Microbe 12: 633–644. 10.1016/j.chom.2012.10.007 23159053 PMC3556742

[39] Zhang F, Landford WN, Ng M, McNatt MW, Bieniasz PD, et al (2011) SIV Nef proteins recruit the AP-2 complex to antagonize Tetherin and facilitate virion release. PLoS Pathog 7: e1002039 10.1371/journal.ppat.1002039 21625568 PMC3098198

[40] Le Tortorec A, Neil SJ (2009) Antagonism to and intracellular sequestration of human tetherin by the human immunodeficiency virus type 2 envelope glycoprotein. J Virol 83: 11966–11978. 10.1128/JVI.01515-09 19740980 PMC2772693

[41] Serra-Moreno R, Zimmermann K, Stern LJ, Evans DT (2013) Tetherin/BST-2 antagonism by Nef depends on a direct physical interaction between Nef and tetherin, and on clathrin-mediated endocytosis. PLoS Pathog 9: e1003487 10.1371/journal.ppat.1003487 23853598 PMC3708871

[42] Lau D, Kwan W, Guatelli J (2011) Role of the endocytic pathway in the counteraction of BST-2 by human lentiviral pathogens. J Virol 85: 9834–9846. 10.1128/JVI.02633-10 21813615 PMC3196438

[43] Sauter D, Unterweger D, Vogl M, Usmani SM, Heigele A, et al (2012) Human tetherin exerts strong selection pressure on the HIV-1 group N Vpu protein. PLoS Pathog 8: e1003093 10.1371/journal.ppat.1003093 23308067 PMC3534379

[44] Kluge SF, Mack K, Iyer SS, Pujol FM, Heigele A, et al (2014) Nef proteins of epidemic HIV-1 group O strains antagonize human tetherin. Cell Host Microbe 16: 639–650. 10.1016/j.chom.2014.10.002 25525794 PMC4274627

[45] Tavano B, Galao RP, Graham DR, Neil SJ, Aquino VN, et al (2013) Ig-like transcript 7, but not bone marrow stromal cell antigen 2 (also known as HM1.24, tetherin, or CD317), modulates plasmacytoid dendritic cell function in primary human blood leukocytes. J Immunol 190: 2622–2630. 10.4049/jimmunol.1202391 23401591 PMC3672947

[46] Megjugorac NJ, Jacobs ES, Izaguirre AG, George TC, Gupta G, et al (2007) Image-based study of interferongenic interactions between plasmacytoid dendritic cells and HSV-infected monocyte-derived dendritic cells. Immunol Invest 36: 739–761. 18161527 10.1080/08820130701715845

[47] Sato K, Misawa N, Fukuhara M, Iwami S, An DS, et al (2012) Vpu augments the initial burst phase of HIV-1 propagation and downregulates BST2 and CD4 in humanized mice. J Virol 86: 5000–5013. 10.1128/JVI.07062-11 22357275 PMC3347374

[48] Dave VP, Hajjar F, Dieng MM, Haddad E, Cohen EA (2013) Efficient BST2 antagonism by Vpu is critical for early HIV-1 dissemination in humanized mice. Retrovirology 10: 128 10.1186/1742-4690-10-128 24195843 PMC4226203

[49] Haase AT (2010) Targeting early infection to prevent HIV-1 mucosal transmission. Nature 464: 217–223. 10.1038/nature08757 20220840

[50] Bego MG, Dube M, Mercier J, Cohen EA (2009) Effect of calcium-modulating cyclophilin ligand on human immunodeficiency virus type 1 particle release and cell surface expression of tetherin. J Virol 83: 13032–13036. 10.1128/JVI.01786-09 19793820 PMC2786830

[51] Dube M, Roy BB, Guiot-Guillain P, Binette J, Mercier J, et al (2010) Antagonism of tetherin restriction of HIV-1 release by Vpu involves binding and sequestration of the restriction factor in a perinuclear compartment. PLoS Pathog 6: e1000856 10.1371/journal.ppat.1000856 20386718 PMC2851737

[52] Buchacher A, Predl R, Strutzenberger K, Steinfellner W, Trkola A, et al (1994) Generation of human monoclonal antibodies against HIV-1 proteins; electrofusion and Epstein-Barr virus transformation for peripheral blood lymphocyte immortalization. AIDS Res Hum Retroviruses 10: 359–369. 7520721 10.1089/aid.1994.10.359

[53] Trkola A, Purtscher M, Muster T, Ballaun C, Buchacher A, et al (1996) Human monoclonal antibody 2G12 defines a distinctive neutralization epitope on the gp120 glycoprotein of human immunodeficiency virus type 1. J Virol 70: 1100–1108. 8551569 10.1128/jvi.70.2.1100-1108.1996 PMC189917

[54] Mascola JR, Lewis MG, Stiegler G, Harris D, VanCott TC, et al (1999) Protection of Macaques against pathogenic simian/human immunodeficiency virus 89.6PD by passive transfer of neutralizing antibodies. J Virol 73: 4009–4018. 10196297 10.1128/jvi.73.5.4009-4018.1999 PMC104180

[55] Etemad-Moghadam B, Sun Y, Nicholson EK, Karlsson GB, Schenten D, et al (1999) Determinants of neutralization resistance in the envelope glycoproteins of a simian-human immunodeficiency virus passaged in vivo. J Virol 73: 8873–8879. 10482646 10.1128/jvi.73.10.8873-8879.1999 PMC112913

[56] Crawford JM, Earl PL, Moss B, Reimann KA, Wyand MS, et al (1999) Characterization of primary isolate-like variants of simian-human immunodeficiency virus. J Virol 73: 10199–10207. 10559336 10.1128/jvi.73.12.10199-10207.1999 PMC113073

[57] Ablashi DV, Berneman ZN, Kramarsky B, Whitman J Jr., Asano Y, et al (1995) Human herpesvirus-7 (HHV-7): current status. Clin Diagn Virol 4: 1–13. 15566823 10.1016/0928-0197(95)00005-s

[58] Keele BF, Giorgi EE, Salazar-Gonzalez JF, Decker JM, Pham KT, et al (2008) Identification and characterization of transmitted and early founder virus envelopes in primary HIV-1 infection. Proc Natl Acad Sci U S A 105: 7552–7557. 10.1073/pnas.0802203105 18490657 PMC2387184

[59] Salazar-Gonzalez JF, Bailes E, Pham KT, Salazar MG, Guffey MB, et al (2008) Deciphering human immunodeficiency virus type 1 transmission and early envelope diversification by single-genome amplification and sequencing. J Virol 82: 3952–3970. 10.1128/JVI.02660-07 18256145 PMC2293010

[60] Lee HY, Giorgi EE, Keele BF, Gaschen B, Athreya GS, et al (2009) Modeling sequence evolution in acute HIV-1 infection. J Theor Biol 261: 341–360. 10.1016/j.jtbi.2009.07.038 19660475 PMC2760689

[61] Salazar-Gonzalez JF, Salazar MG, Keele BF, Learn GH, Giorgi EE, et al (2009) Genetic identity, biological phenotype, and evolutionary pathways of transmitted/founder viruses in acute and early HIV-1 infection. J Exp Med 206: 1273–1289. 10.1084/jem.20090378 19487424 PMC2715054

[62] Cohen GB, Gandhi RT, Davis DM, Mandelboim O, Chen BK, et al (1999) The selective downregulation of class I major histocompatibility complex proteins by HIV-1 protects HIV-infected cells from NK cells. Immunity 10: 661–671. 10403641 10.1016/s1074-7613(00)80065-5

[63] Klimkait T, Strebel K, Hoggan MD, Martin MA, Orenstein JM (1990) The human immunodeficiency virus type 1-specific protein vpu is required for efficient virus maturation and release. J Virol 64: 621–629. 2404139 10.1128/jvi.64.2.621-629.1990 PMC249152

[64] Westervelt P, Henkel T, Trowbridge DB, Orenstein J, Heuser J, et al (1992) Dual regulation of silent and productive infection in monocytes by distinct human immunodeficiency virus type 1 determinants. J Virol 66: 3925–3931. 1533883 10.1128/jvi.66.6.3925-3931.1992 PMC241183

[65] Trowitzsch S, Bieniossek C, Nie Y, Garzoni F, Berger I (2010) New baculovirus expression tools for recombinant protein complex production. J Struct Biol 172: 45–54. 10.1016/j.jsb.2010.02.010 20178849

[66] Bego MG, Mercier J, Cohen EA (2012) Virus-activated interferon regulatory factor 7 upregulates expression of the interferon-regulated BST2 gene independently of interferon signaling. J Virol 86: 3513–3527. 10.1128/JVI.06971-11 22301143 PMC3302510

[67] Dube M, Roy BB, Guiot-Guillain P, Mercier J, Binette J, et al (2009) Suppression of Tetherin-restricting activity upon human immunodeficiency virus type 1 particle release correlates with localization of Vpu in the trans-Golgi network. J Virol 83: 4574–4590. 10.1128/JVI.01800-08 19244337 PMC2668474

