# Supplementary figures and images for: Vpu Exploits the Cross-Talk between BST2 and the ILT7 Receptor to Suppress Anti-HIV-1 Responses by Plasmacytoid Dendritic Cells
Source: PLoS Pathog. 2015 Jul 14;11(7):e1005024. doi: 10.1371/journal.ppat.1005024 (PMC4501562; doi:10.1371/journal.ppat.1005024)

Fig. S1

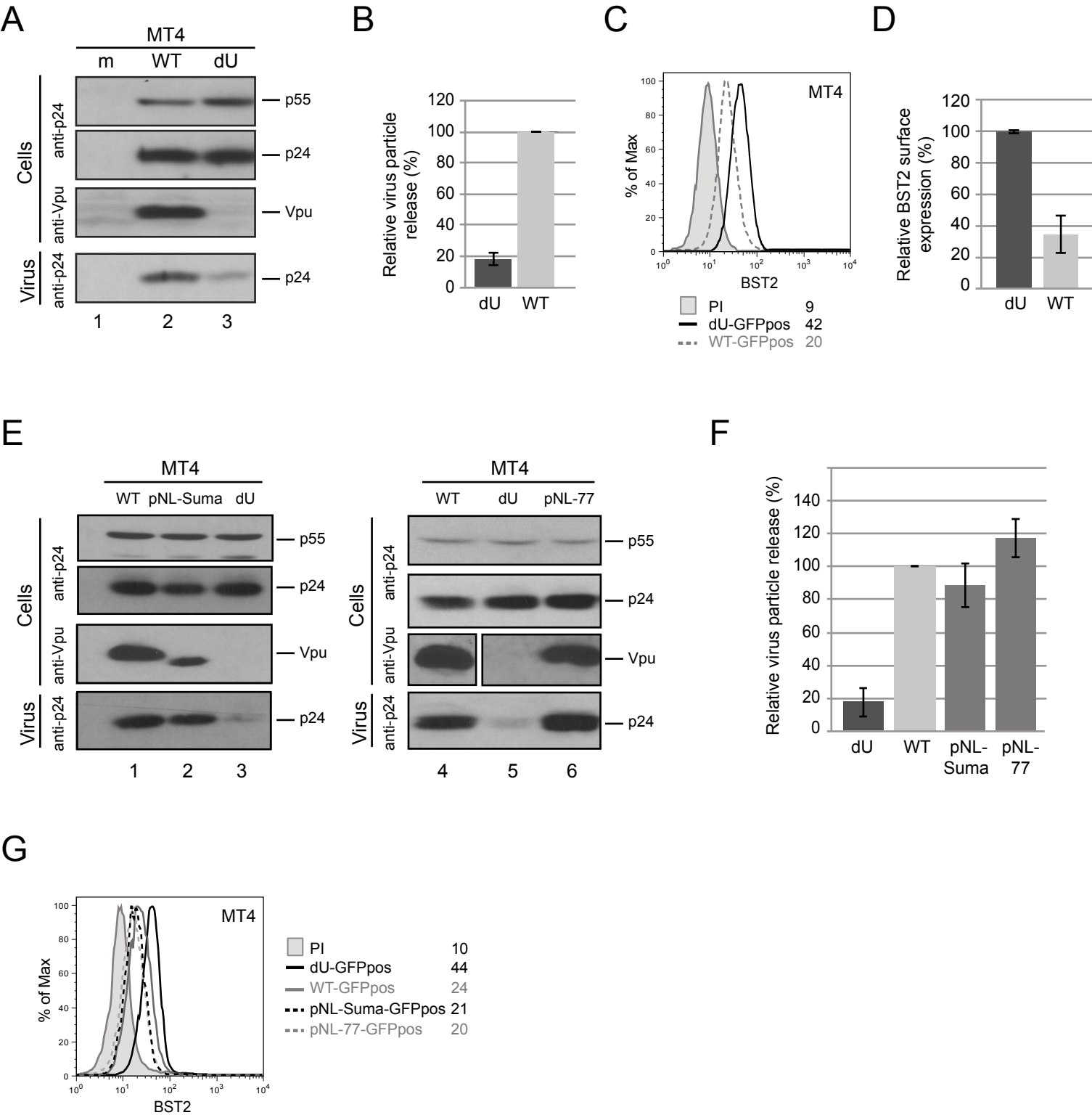

Supplement: S1 Fig — (A-D) MT4 cells were mock-infected (m) or infected with GFP-marked NL4.3 (WT or dU) as indicated. (A) Cells and virion-containing supernatants were analyzed for the presence of Gag proteins and Vpu by Western blot, 48 hours post infection (hpi), as indicated. (B) Relative virus particle release efficiency was calculated as described in Materials and Methods and normalized to the value obtained with the WT virus, which was set at 100% (n = 3). (C) Flow cytometry analysis of surface BST2 in GFP-positive MT4 cells infected with WT (dashed grey histogram) or dU (solid black histogram), 48 hpi. Mean fluorescence intensity (MFI) values are indicated for each sample (staining using pre-immune rabbit serum, PI, shaded grey histograms). (D) Relative BST2 surface expression after infection with the indicated HIV viruses (n = 4). Percentage MFI were calculated relative to dU HIV-producing cells (100%). (E-G) MT4 cells were infected with GFP-marked NL4.3 virus lacking Vpu (dU) or encoding either NL4.3 Vpu (WT), T/F Suma Vpu (pNL-Suma) or T/F CH077 Vpu (pNL-77). (E) Cells and virion-containing supernatants were analyzed by Western blot as described in panel A. Note that detection of T/F CHO77 Vpu required a longer exposure since rabbit polyclonal anti-BST2 Abs were inefficient at recognizing this Vpu variant. (F) Relative virus particle release efficiency was determined as described in panel B (n = 2). (G) Surface BST2 expression was evaluated by flow cytometry 48 hpi as described in panel C. Error bars represent standard deviations (SD). (PDF) [file ppat.1005024.s001.pdf]

**Fig. S2****A**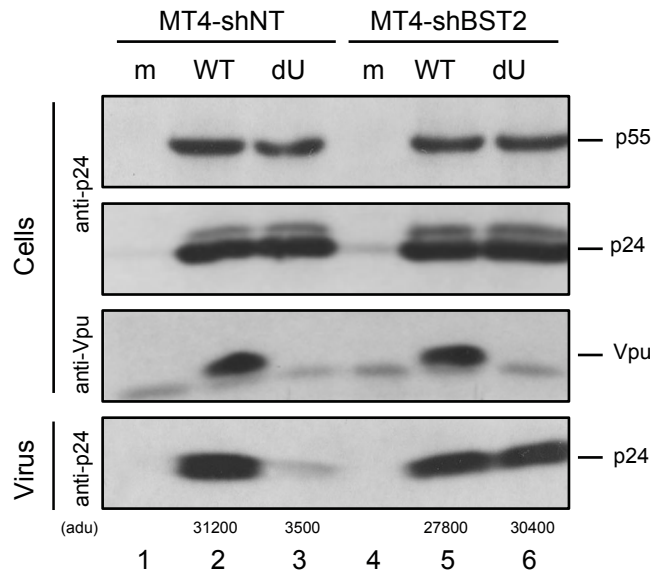**B**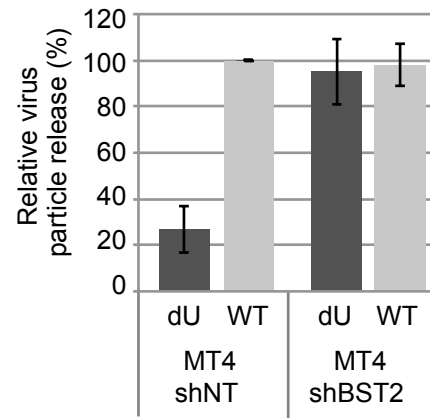**C**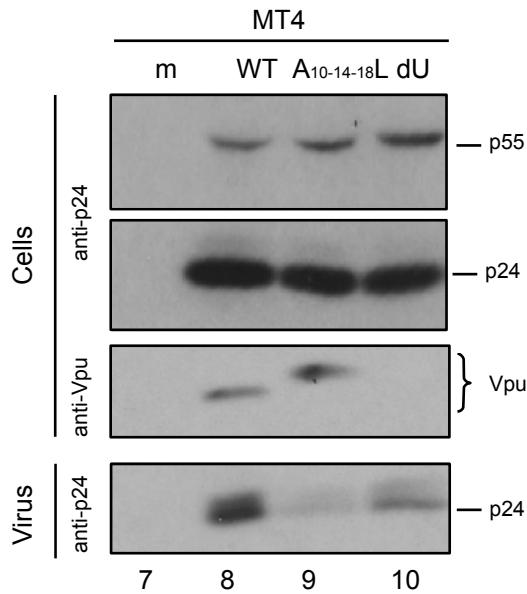**D**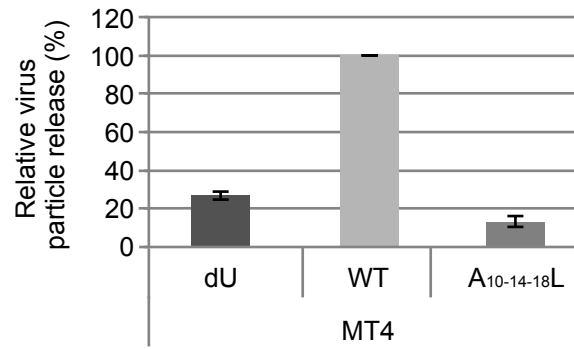**E**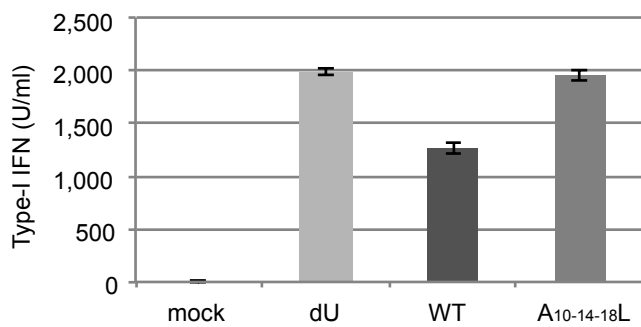**F**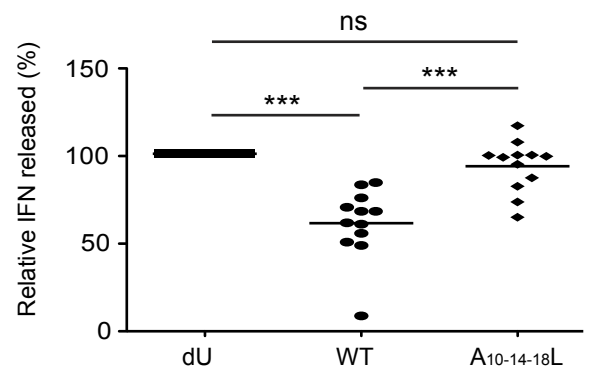

Supplement: S2 Fig — (A) Cells and virion-containing supernatants were analyzed by Western blot as described in S1 Fig. The absolute amounts of virus released in each condition was estimated by densitometry scanning of the virion-associated p24 signal and is indicated under the blot as arbitrary densitometric unit (adu). (B) Relative virus particle release efficiency was determined as described in S1 Fig (n = 3). (C-F) MT4 cells were mock-infected or infected with GFP-marked NL4.3 WT, dU or VpuA10-14-18L TM mutant viruses. (C) Cells and virion-containing supernatants were analyzed by western blot as described in S1 Fig. (D) Relative virus particle release efficiency was determined as described in S1 Fig (n = 3). (E-F) The indicated MT4 donor cells were co-cultured with PBMCs. After 24 h, levels of IFN-I released in supernatants were measured. A representative example of absolute levels (E) or relative percentages (F) of IFN-I production after co-culture of infected MT4 cells with PBMCs are shown. The amount of IFN-I released by PBMCs in contact with dU HIV-infected cells was set at 100% (n = 12). Repeated measures ANOVA with Bonferroni’s multiple comparison tests was used (*** p<0.001, ns not significant (p>0.05)). Error bars represent standard deviations (SD). (PDF) [file ppat.1005024.s002.pdf]

Fig. S3

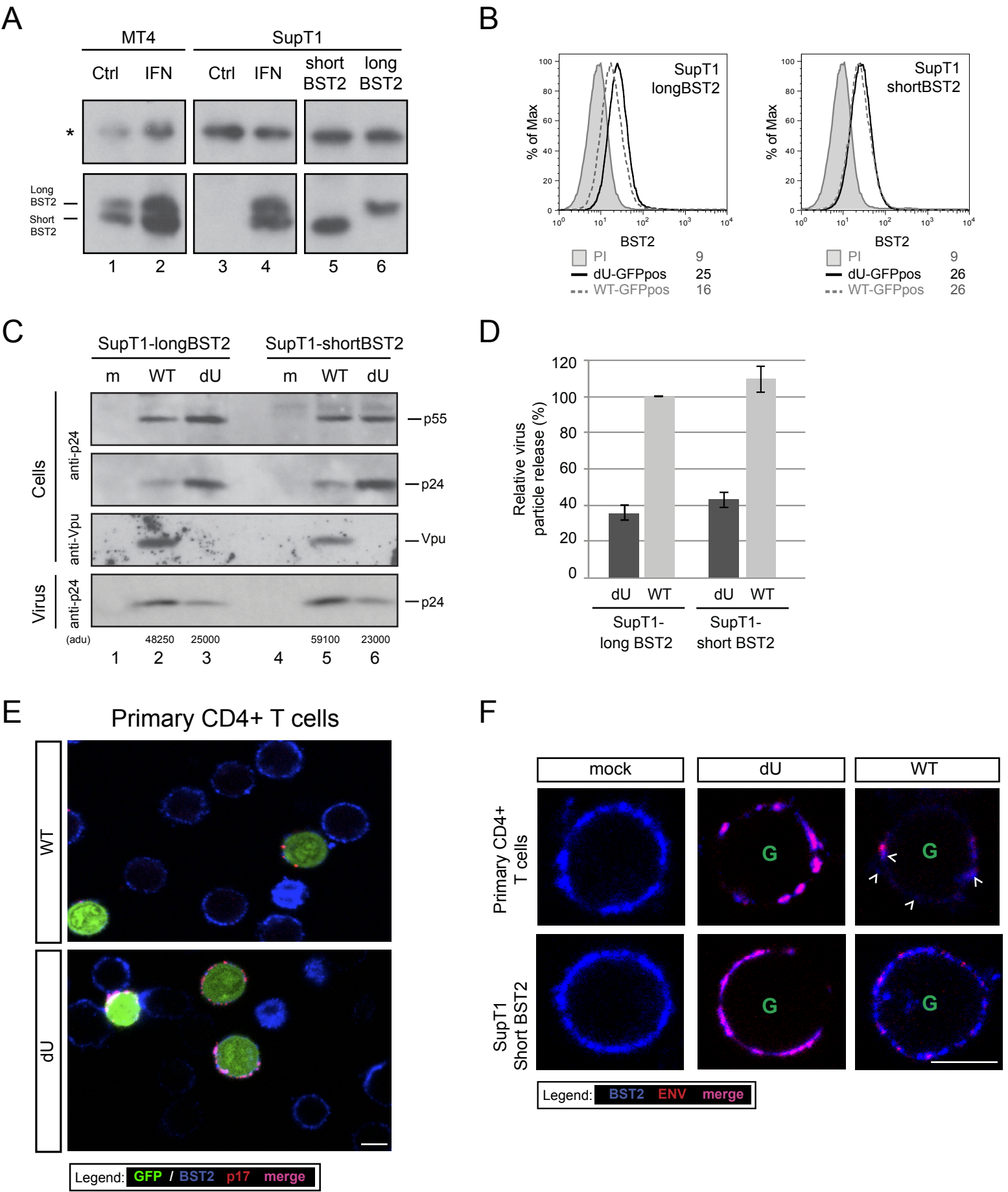

Supplement: S3 Fig — (A) BST2 from SupT1 cells expressing either long or short isoforms was immunoprecipitated, treated with PNGase and analyzed by Western blot. As controls, BST2 from IFN-treated and untreated SupT1 and MT4 cells were similarly analyzed. * represent the Ab heavy chain and was used as loading control. (B-D) SupT1-shortBST2 and SupT1-longBST2 cells were mock-infected (m) or infected with NL4.3-GFP WT or dU viruses. (B) Surface BST2 expression was evaluated by flow cytometry 48 hpi, as described in S1 Fig. (C) Cells and virion-containing supernatants were analyzed by western blot as described in S1 Fig. The absolute amount of virus released in each condition was estimated by densitometry scanning of the virion-associated p24 signal and is indicated under the blot as arbitrary densitometric unit (adu). (D) Relative virus particle release efficiency was determined as described in S1 Fig (n = 3). HIV-1 WT release efficiency in SupT1-longBST2 was set at 100%. Error bars represent standard deviations (SD). (E-F) Primary CD4+ T cells and SupT1-shortBST2 cells were mock-infected (mock) or infected with VSV-G-pseudotyped NL4.3-Ada-GFP WT or dU viruses. (E) Infected primary CD4+ T cells were stained with anti-BST2 Abs (blue), fixed, permeabilized and then sequentially stained with anti-p17 Abs (red). A representative example of multiple cells is shown. (F) Infected primary CD4+ T cells and SupT1-shortBST2 cells were stained with anti-BST2 Abs (blue) and 2G12 anti-Env Abs (red). A representative example is shown. White bar = 10 μm. (PDF) [file ppat.1005024.s003.pdf]

Fig. S4

A

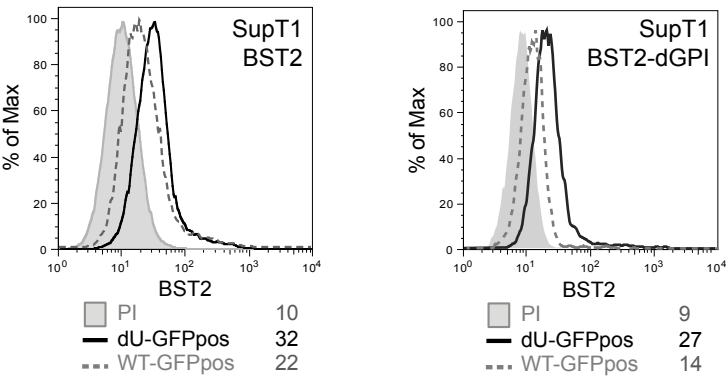

B

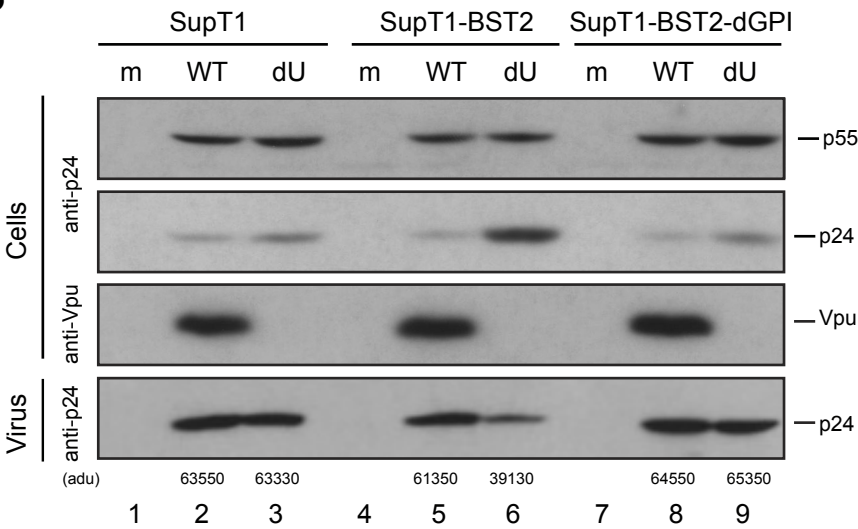

C

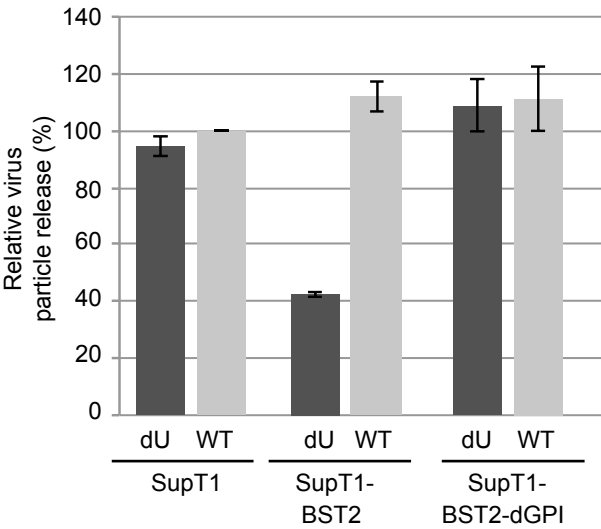

Supplement: S4 Fig — SupT1-Empty, SupT1-BST2 and SupT1-BST2-dGPI cells were mock-infected or infected with GFP-marked NL4.3 WT or dU viruses. (A) Surface BST2 expression was evaluated by flow cytometry 48 hpi as described in S1 Fig. (B) Cells and virion-containing supernatants were analyzed by western blot as described in S1 Fig. The absolute amounts of virus released in each condition was estimated by densitometry scanning of the virion-associated p24 signal and is indicated under the blot as arbitrary densitometric unit (adu). (C) Relative virus particle release efficiency was determined as described in S1 Fig (n = 3). HIV-1 WT release efficiency in SupT1-BST2 was set at 100%. Error bars represent standard deviations (SD). (PDF) [file ppat.1005024.s004.pdf]
