# Supplementary material for: Vpu Exploits the Cross-Talk between BST2 and the ILT7 Receptor to Suppress Anti-HIV-1 Responses by Plasmacytoid Dendritic Cells
Source: PLoS Pathog. 2015 Jul 14;11(7):e1005024. doi: 10.1371/journal.ppat.1005024 (PMC4501562; doi:10.1371/journal.ppat.1005024)
Supplement: S5 Fig — SupT1 (no BST2), SupT1-longBST2 (long BST2) and SupT1-shortBST2 (short BST2) cells were mock-infected or infected with GFP-marked NL4.3 WT or T/F CH077 viruses for 48 h prior to co-culture with PBMCs. After 24 h of co-culture, levels of IFN-I released in supernatants were measured. A representative example of absolute levels (A) or relative percentages (B) of IFN-I detected after co-culture of WT or dU HIV-1-infected SupT1 donor cells with PBMCs. The amount of IFN-I released by PBMCs in contact with NL4.3- or CH077-infected SupT1-BST2 cells in the absence of BST2 was set at 100%. Repeated measures ANOVA with Bonferroni’s multiple comparison test was used (n >3). Error bars represent SD. ns- not significant. (PDF) [file ppat.1005024.s005.pdf]

Fig. S5

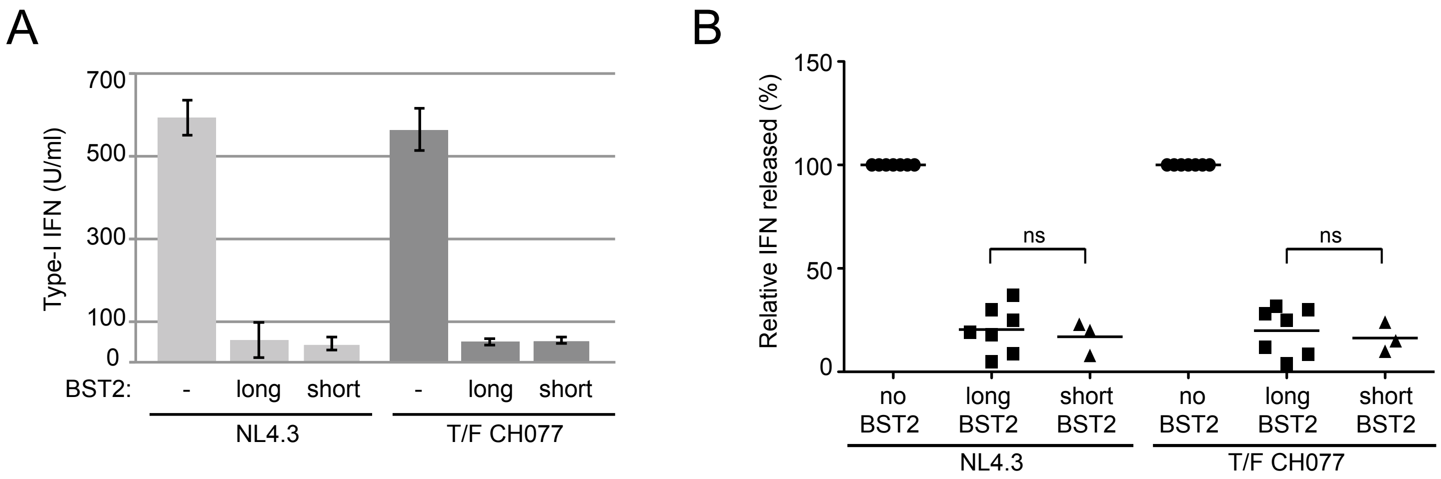

Repeated measures ANOVA with Bonferroni's multiple comparison test was used. Error bars represent SD.
